# Systematic Review: Anesthetic Protocols and Management as Confounders in Rodent Blood Oxygen Level Dependent Functional Magnetic Resonance Imaging (BOLD fMRI)—Part B: Effects of Anesthetic Agents, Doses and Timing

**DOI:** 10.3390/ani11010199

**Published:** 2021-01-15

**Authors:** Aline R. Steiner, Frédérik Rousseau-Blass, Aileen Schroeter, Sonja Hartnack, Regula Bettschart-Wolfensberger

**Affiliations:** 1 Section of Anaesthesiology, Department of Clinical and Diagnostic Services, Vetsuisse Faculty, University of Zurich, 8057 Zurich, Switzerland; rbettschart@vetclinics.uzh.ch; 2 Department of Clinical Sciences, Faculty of Veterinary Medicine, Université de Montréal, Saint-Hyacinthe, QC J2S 2M2, Canada; frederik.rousseau-blass@hotmail.com; 3 Institute for Biomedical Engineering, University and ETH Zurich, 8093 Zurich, Switzerland; schroeter@biomed.ee.ethz.ch; 4 Section of Epidemiology, Vetsuisse Faculty, University of Zurich, 8057 Zurich, Switzerland; sonja.hartnack@access.uzh.ch

**Keywords:** BOLD fMRI, anesthetic protocol, anesthesia regime, isoflurane, medetomidine, α-chloralose, validity, rat, mouse

## Abstract

**Simple Summary:**

To understand brain function in rats and mice functional magnetic resonance imaging of the brain is used. With this type of “brain scan” regional changes in blood flow and oxygen consumption are measured as an indirect surrogate for activity of brain regions. Animals are often anesthetized for the experiments to prevent stress and blurred images due to movement. However, anesthesia may alter the measurements, as blood flow within the brain is differently affected by different anesthetics, and anesthetics also directly affect brain function. Consequently, results obtained under one anesthetic protocol may not be comparable with those obtained under another, and/or not representative for awake animals and humans. We have systematically searched the existing literature for studies analyzing the effects of different anesthesia methods or studies that compared anesthetized and awake animals. Most studies reported that anesthetic agents, doses and timing had an effect on functional magnetic resonance imaging results. To obtain results which promote our understanding of brain function, it is therefore essential that a standard for anesthetic protocols for functional magnetic resonance is defined and their impact is well characterized.

**Abstract:**

In rodent models the use of functional magnetic resonance imaging (fMRI) under anesthesia is common. The anesthetic protocol might influence fMRI readouts either directly or via changes in physiological parameters. As long as those factors cannot be objectively quantified, the scientific validity of fMRI in rodents is impaired. In the present systematic review, literature analyzing in rats and mice the influence of anesthesia regimes and concurrent physiological functions on blood oxygen level dependent (BOLD) fMRI results was investigated. Studies from four databases that were searched were selected following pre-defined criteria. Two separate articles publish the results; the herewith presented article includes the analyses of 83 studies. Most studies found differences in BOLD fMRI readouts with different anesthesia drugs and dose rates, time points of imaging or when awake status was compared to anesthetized animals. To obtain scientifically valid, reproducible results from rodent fMRI studies, stable levels of anesthesia with agents suitable for the model under investigation as well as known and objectively quantifiable effects on readouts are, thus, mandatory. Further studies should establish dose ranges for standardized anesthetic protocols and determine time windows for imaging during which influence of anesthesia on readout is objectively quantifiable.

## 1. Introduction

Functional magnetic resonance imaging (fMRI) is commonly used in rat and mice models to study mechanisms underlying physiological as well as pathological brain functions [1,2,3]. 

The present review concentrates on blood oxygen level dependent (BOLD) fMRI. Assuming that neuronal activity is coupled to local blood flow—although this may not apply to all brain regions [4], and the direction of correlations may vary [5]—this diagnostic imaging technique assesses neuronal activation based on measured changes in blood oxygen (O_2_) levels [6]: If certain brain regions are neuronally activated, arterioles and eventually also capillaries are dilated and more fully oxygenated blood flows in [7]. When locally, in activated areas, O_2_ supply exceeds O_2_ demand, venous oxygenation increases. Therefore, the proportion of deoxygenated hemoglobin to oxygenated hemoglobin is reduced. Changes in the ratio of deoxy- to oxyhemoglobin content per unit of brain determine changes in BOLD signal. Cerebral blood volume (CBV), cerebral metabolic rate of oxygen (CMRO_2_) and cerebral blood flow (CBF) are determinants of deoxyhemoglobin content [8]. Therefore, without an underlying change in neural activity, changes in CMRO_2_, CBF or CBV may affect BOLD signal intensity.

To avoid movement artefacts animals must be immobilized for fMRI. Although imaging of conscious restrained animals is applied [9], to date the majority of studies in rats and mice achieve immobilization by general anesthesia or sedation (in the following termed “anesthesia”). 

A systematic review of anesthetic protocols used for pharmacological fMRI (phMRI) found however 73 different combinations of anesthetic agents, doses and ventilatory gas mixtures in 126 publications, and that publications using identical protocols typically originated from the same authors [10].

This is problematic for several reasons. First, anesthetics per definitionem alter brain functions as observable on a macroscopic level, and it is believed that this altered state of functionality results from reduced information integration [11]. Based on local as well as global electrophysiological measurements, different anesthetics however differently affect neuronal spontaneous and evoked activity [12].

Second, several aspects of neurovascular coupling are affected: CMRO_2_ is reduced in comparison to the awake state [9], anesthetics may modulate signal cascades responsible for neurovascular coupling [13,14] and hemodynamics are altered under anesthesia, which is evident as slower CBF and CBV reactions to stimulation with lower amplitudes in comparison to awake animals [9].

Finally, anesthetics induce agent-specific changes in systemic physiology, notably cardiovascular and respiratory parameters, which in turn may affect cerebral hemodynamics [15]. 

Considering the various mechanisms by which specific anesthetic protocols or anesthetic management might influence BOLD fMRI readouts and introduce bias, it is questionable if and how valid results can be gathered from animals under general anesthesia. 

The aim of the present review was to provide direct evidence for the range and extent of anesthetic regimen-related differences in BOLD fMRI readouts. A systematic search was initiated to find rat and mouse studies that (a) investigated effects of changes in systemic physiological parameters on BOLD fMRI results and/or (b) directly compared BOLD fMRI results obtained with the same imaging protocol under different anesthetic agents, at different doses of the same agent, at different timepoints relative to induction of anesthesia, and/or in anesthetized versus awake animals. Two separate analyses were then performed in parallel to identify (a) effects of systemic physiological parameter changes regardless of the anesthetics used in those studies, and (b) to identify differences between different states of anesthesia (i.e., between anesthetic agents, doses, timepoints of imaging or between awake and anesthetized imaging) regardless of eventual physiological parameter changes. 

Part a [15] of this review showed that changes in physiological parameters confound results of BOLD fMRI studies when undetected—in particular arterial partial pressure of carbon dioxide (CO_2_), blood oxygenation and arterial blood pressures. Establishment of standards for anesthetic management and monitoring was claimed. Here, in part b, effects of doses of anesthetic agents, time points of imaging and agent selection are presented, which were found in most of the included studies. It is concluded that a selection of validated anesthetic protocols should be established.

## 2. Materials and Methods 

For a detailed description of the materials and methods used see part a of this review [15]. Preparation, performance and reporting of this systematic review followed the SYRCLE (SYstematic Review Centre for Laboratory animal Experimentation, Radboud University, Nijmegen, The Netherlands) protocol for systematic reviews of animal intervention studies [16]. Inclusion and exclusion criteria are specified in Table 1. 

## 3. Results

Results of the systematic search, including a PRISMA flow chart as well as detailed results of the risk of bias assessment of all included studies, are reported in part a of the review [15]. 

This part of the review reports synthesized findings of the 83 references which investigated effects of different states of anesthesia on BOLD fMRI readouts in rats (70 references based on 62 datasets) and mice (13 references based on 11 datasets). Reference counts were higher than datasets because re-analysis of an existing data set was permitted in our inclusion criteria. Overlap with references analyzed in part a of this review is small; only 8 rat and 3 mouse references additionally addressed effects of physiological parameter changes. All references scored as having a high risk of bias. 

An overview of the agents and aspects (doses; timepoints; versus awake) compared in included references is provided in Supplementary Materials Table S1. Additionally, a separate set of tables summarizes the findings of each study and specifies the doses of anesthetics used (Supplementary Materials Tables S2–S6); for those tables, studies were grouped by species and type of BOLD fMRI outcome. Animal characteristics are summarized in Supplementary Materials Document S7.

In the following, main findings of comparisons between anesthetic doses, time points of imaging, pairwise comparison of anesthetics and comparison of anesthetized with awake imaging are highlighted. Findings for medetomidine and its active enantiomer dexmedetomidine were summarized, as both drugs have the same sedative and cardiovascular effects and share almost identical pharmacokinetics when dexmedetomidine is administered at 50% of the medetomidine dose [17,18].

### 3.1. Effects of Anesthetic Dose

**Figure 1:**
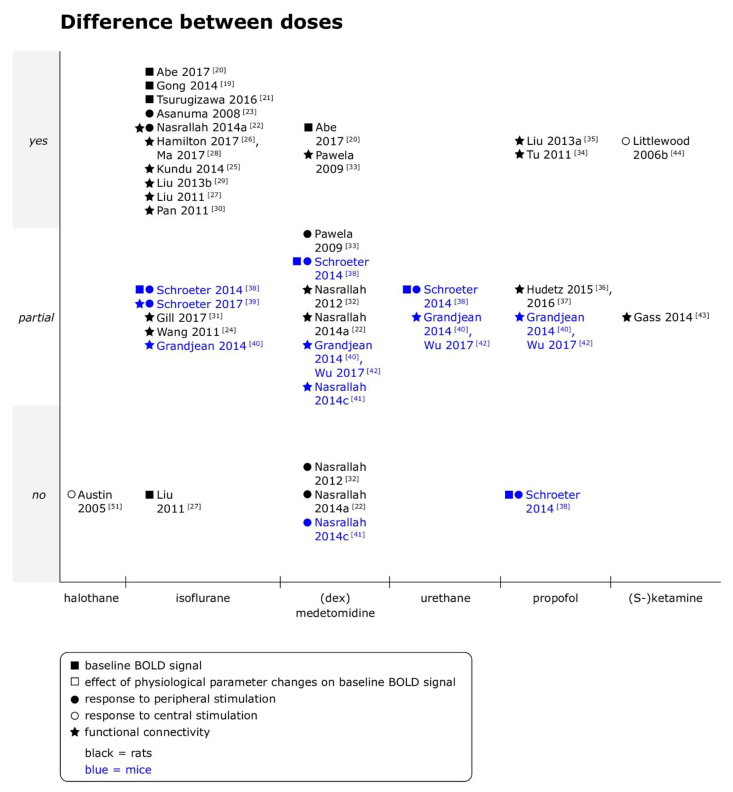
Effects of anesthetic doses on baseline blood oxygen level dependent (BOLD) signal, responses to peripheral and central stimulation and functional connectivity in rats and mice. Yes = differences between doses were clearly observed; partial = differences between doses were observed for some outcomes, analyses or animals, but not consistently, or quantitative differences were reported, but statistical significance unclear; and no = no differences between doses were observed. When datasets were re-analyzed, findings were pooled and one data point per specific comparison (i.e., per anesthetic per type of functional magnetic resonance imaging (fMRI)) displayed. (dex)medetomidine = medetomidine or dexmedetomidine was studied. (S-)ketamine = ketamine or S-ketamine was studied. Not all references are discussed in the text [43,44], but a summary of the main findings as well as the doses of anesthetics are provided for references with the symbols ■ or □ in Supplementary Materials Tables S2 and S5, with symbols ● or ○ in Supplementary Materials Tables S3 and S5, and for references with symbol ★ in Supplementary Materials Tables S4 and S6.

Effects of anesthetic dose on BOLD fMRI outcomes were investigated in 24 of the included datasets for 43 combinations of anesthetic agent and type of fMRI (e.g., dose effects of isoflurane on response to peripheral stimulation, see Figure 1), as studies commonly investigated effects of several anesthetic agents and/or on different types of fMRI. Only in 6 out of 43 comparisons the dose of anesthetic had no influence on BOLD fMRI outcomes. Isoflurane (18 comparisons) and medetomidine (11 comparisons) were most extensively studied, followed by propofol (5 comparisons).

Rats

Under isoflurane, baseline BOLD signal increased from 1.8% to 3.5% and from 1.5% to 2.5% [19,20] or followed an inverse U-shape between 1.5% and 3.0% [21]. There was no trend for stimulation and resting state fMRI (rsfMRI) to differ in the sensitivity to dose effects. Responses to peripheral electrical stimulation were of lower amplitude when isoflurane concentrations were increased in a range of 1% to 3% [22]. Expected responses to subcutaneous (sc) formalin injection in rats were only observed at 1.0% and 1.2% within a range of 0.6% to 2.0% isoflurane [23]. In rsfMRI, effects on various outcomes were investigated. Taken together, the included studies indicate monotonic decrease of information content [24,25,26] as well as functional connectivity (fc) strength between multiple “units” (seeds, voxels, regions of interest (ROI)) when higher isoflurane concentrations were used [24,27,28], and non-monotonic dose effects on low frequency BOLD signal fluctuations [22,24,27,29], spatial extent of fc [24,27,29], interhemispheric fc strength [22,24,27,30], dynamic fc [28] and measures of network analysis [31].

Under medetomidine, baseline BOLD signal intensity was reduced at higher doses (0.3 versus 0.1 mg/kg/h intravenously (iv) after a 0.05 mg/kg sc bolus [20]). Interestingly, three studies assessing both responses to stimulation and fc found more pronounced effects of medetomidine doses on rsfMRI outcomes than on responses to stimulation [22,32,33]. Responses to peripheral electrical stimulation were not affected by different medetomidine infusion rates in two studies (01., 0.2 or 0.3 mg/kg/h intraperitoneal (ip) after a 0.05 mg/kg ip bolus in both studies [22,32]), but the dependence of responses on stimulation frequency partially depended on the medetomidine infusion rate when imaging was continued for more than 2 h (0.1, 0.15, 0.2 or 0.3 mg/kg/h iv after 2 h of 0.1 mg/kg/h iv [33]). When it comes to rsfMRI, higher doses of medetomidine were consistently associated with reduced strength and/or occurrence of interhemispheric connectivity between homotopic regions, but unlike for isoflurane, amplitude of signal fluctuations or total power did not differ between doses [22,32]. An interaction between time and doses was found for pairwise correlation coefficient matrices of ROI belonging to the sensorimotor network: If infusion rate was kept constant for more than 2 h, correlation coefficients typically (significantly) decreased beyond that time point, whereas an increase of infusion rate after 2 h lead to unchanged and occasionally increased correlation coefficients [33]. 

Dose effects of propofol were in rats only for rsfMRI investigated. While increasing the dose of a single bolus from 80 to 160 mg/kg ip, or increasing the rate of a constant rate infusion (CRI) from 20 to 40 mg/kg/h reduced spatial extent of fc maps [34,35], complex, non-monotonic patterns of dose-dependence were found in whole brain as well as regional fc when dose rates were further increased up to 100 mg/kg/h [35]. Temporal variation of regional homogeneity (ReHo), a measure of local connectivity, but not average ReHo of selected voxels was reduced at 40 versus 20 mg/kg/h, as well as the number of unique brain states and the non-redundant information content of the BOLD signal [36,37].

Mice

Under isoflurane, stronger baseline BOLD signal fluctuations and higher amplitudes of responses to peripheral electrical stimulation were observed at 1.0% than at 1.5% isoflurane, with the caveat that location of the responses in that study was unspecific at both concentrations and suspected by the authors to be of cardiovascular origin [38]. A follow-up study observed that the lack of specificity of the response, indicated by the absent difference in peak signal change between ipsi- and contralateral primary somatosensory cortex (S1), did not depend on isoflurane concentrations within the same range [39]. For rsfMRI however, spatially more specific correlations with a seed in sensory cortex were observed at 1.0 than at 1.5% [40]. 

Conflicting results were reported for dose effects of medetomidine on responses to peripheral electrical stimulation: While one study reported stronger baseline BOLD signal fluctuations and higher peak signal changes under of 0.1 than 0.2 mg/kg/h medetomidine iv after a bolus of 0.05 or 0.1 mg/kg iv [38], another study found no difference in responses to stimulation between CRI rates of 0.1, 0.3 and 0.6 mg/kg/h ip after a bolus of 0.3 mg/kg ip [41]. In rsfMRI, lower doses of medetomidine were consistently associated with higher fc values in seed-based interhemispheric connectivity [40,41] over the same range of doses, but no differences were observed in ReHo in several ROI [42]. Frequency analysis found no significant difference in mean peak frequency [40,41]; and approximate entropy of the signal in a seed in S1 did also not differ between doses [40].

In contrast to isoflurane and medetomidine, no difference in baseline BOLD signal fluctuation or peak signal change in response to peripheral electrical stimulation was observed between two doses of propofol ([38]; 30 mg/kg iv, followed by 120 and later 150 mg/kg/h iv versus 45 mg/kg iv, followed by 187 and later 225 mg/kg/h iv), and dose effects on rsfMRI were not reported beyond a general note of higher fc strengths at the lower of the same two doses [40].

Summary

Overall, dose effects were commonly reported and the observed trends not necessarily monotonic when multiple doses were compared. When data from rats and mice was summarized, dose effects were more pronounced under isoflurane than under medetomidine (no effect found in 1/18 [27] versus 3/11 comparisons [22,32,41]), and under medetomidine, rsfMRI tended to be more susceptible than stimulation fMRI (no dose effect in 0/5 versus 3/5 comparisons [22,32,41]), although not all rsfMRI outcome measures were affected to the same degree. 

### 3.2. Time-Dependent Effects

**Figure 2:**
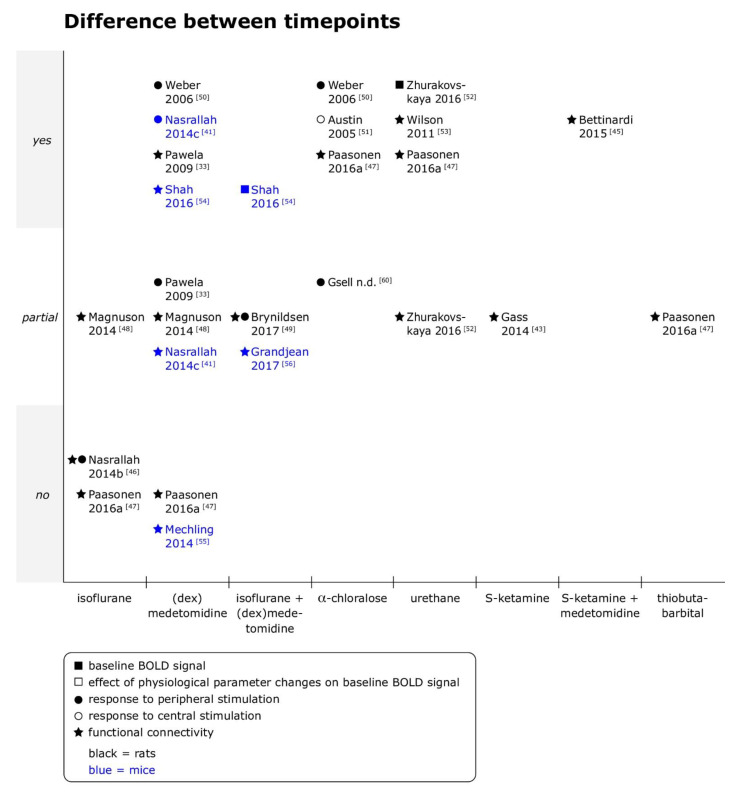
Effects of time points of imaging on baseline BOLD signal, responses to peripheral and central stimulation and functional connectivity in rats and mice. Yes = differences between timepoints were clearly observed; partial = differences between timepoints were observed for some outcomes, analyses or animals, but not consistently, or quantitative differences were reported, but statistical significance unclear; and no = no differences between timepoints were observed. When datasets were re-analyzed, findings were pooled and one data point per specific comparison (i.e., per anesthetic per type of fMRI) displayed. (dex)medetomidine = medetomidine or dexmedetomidine was studied. Not all references are discussed in the text [43,45], but a summary of the main findings as well as the doses of anesthetics are provided for references with the symbols ■ or □ in Supplementary Materials Tables S2 and S5, with symbols ● or ○ in Supplementary Materials Table S3 and S5, and for references with symbol ★ in Supplementary Materials Tables S4 and S6.

Effects of the timepoint of imaging relative to the administration of anesthetics on BOLD fMRI outcomes were investigated in 17 datasets for 28 combinations of anesthetic agent and type of fMRI (see Figure 2). Only 5 of the 28 included comparisons found no time-dependent effects on the specified BOLD fMRI outcomes under the anesthetic studied. 

Time-dependent effects were overall most extensively studied for (dex)medetomidine (nine comparisons), followed by isoflurane, combined isoflurane/(dex)medetomidine, α-chloralose and urethane (four comparisons each).

Rats

The limited data available for inhalant anesthetics, or more precisely isoflurane, did not show time-dependent effects on responses to peripheral electrical stimulation [46], interhemispheric connectivity [46] or average coherence between ROI [47] over observation periods of maximally 1 h at a concentration of 1.3%. However, the duration of previous isoflurane exposure (30 min versus 3 h at 2%) affected rsfMRI results obtained afterwards under a CRI of dexmedetomidine (0.025 mg/kg sc bolus, followed by 0.05 mg/kg/h sc for 80 min, then 0.15 mg/kg/h sc) for up to 3.25 h [48], and effects were seen in various outcome measures, ranging from interhemispheric and whole brain connectivity over spectral characteristics of the signal time course to spatiotemporal dynamics (for details see Supplementary Materials Table S4). Within groups, significant differences between the first and second half of the imaging period were found in spectral characteristics, and a coordinated cortical spatiotemporal dynamic was identified in 50% or more of scans only from a certain timepoint on in both groups. In the “long” isoflurane exposure group, S1 interhemispheric connectivity additionally significantly increased from the first to the second half of the imaging period [48].

Although time-dependent effects from isoflurane and dexmedetomidine are intertwined in that study, it overall suggests that time-dependent effects are present under (dex)medetomidine. This is supported by the observation that fc maps of a seed in orbital frontal cortex were mainly localized around the seed within the first 30 min after bolus administration [49], and spatially more extended in the following 2 h with significant increases of connectivity strength in selected connections, in a combined dexmedetomidine (0.015 mg/kg ip, followed by 0.015 mg/kg/h sc) and isoflurane (0.5%–0.7%) protocol. However, connectivity may also decrease over time: A study using medetomidine alone and without an initial bolus reported that in connectivity matrices of sensorimotor network regions, pairwise correlation coefficients typically (and often significantly) decreased after 2.5 h, unless the infusion rate was at least doubled after 2 h (from 0.1 mg/kg/h iv to 0.2 or 0.3 mg/kg/h iv) [33]. In contrast, another study also using medetomidine alone, but with an initial bolus (0.01 mg/kg iv, followed by 0.1 mg/kg/h iv) did not find a difference in average pairwise coherence between 91 ROI when two timepoints of measurement 1 h apart were compared [47].

Responses to peripheral electrical stimulation were under medetomidine alone only observed around 80 min after bolus administration (0.05 mg/kg sc, followed by 0.1 mg/kg/h sc) [50], and signal intensity of the response in S1 was lower in the first 30 min of measurements than in the following 2 h when dexmedetomidine (0.015 mg/kg ip, followed by 0.015 mg/kg/h sc) was administered together with isoflurane (0.5%–0.75%) [49]. In contrast, a study omitting the initial bolus found no difference over time in signal intensity, but the number of activated voxels became less dependent on stimulation frequency after 2.5 h unless the infusion rate was at least doubled after 2h (from 0.1 mg/kg/h iv to 0.2 or 0.3 mg/kg/h iv) [33].

For α-chloralose, studies reported qualitatively similar findings as for (dex)medetomidine. After the initial bolus, response to peripheral electrical stimulation electrical stimulation was observed only from around 60 min after bolus administration on (50 mg/kg iv, followed by 36 mg/kg iv every 60 min; [50]). Responses to direct electrical stimulation of the motor cortex were stable within the first 2 h after bolus administration (80 mg/kg ip, followed by 30 mg/kg ip 30 min after initial bolus and every 60 min thereafter), and increased in spatial extent as well as in intensity thereafter [51]. Average pairwise coherence between 91 ROI also increased under α-chloralose (60 mg/kg iv, followed by 30 mg/kg iv every 60 min) when measurements were repeated 1h after the first scan [47].

In contrast, under urethane, a distinct cycling pattern was observed, in which phases of lower and higher baseline signal alternated [52] and were associated with fluctuations in the strength of ROI–ROI connectivities [52,53] and more or less extended fc maps [53] at 1.0 and 1.5 g/kg iv and ip, respectively. As under α-chloralose, average pairwise coherence between 91 ROI increased when measurements were repeated 1h after the first scan (1.25 g/kg ip; [47]).

Mice

In mice, time-dependent effects were only investigated for medetomidine and the combination of medetomidine and isoflurane. The baseline BOLD signal was weakest 20 min after administration of a single medetomidine bolus (0.3 mg/kg sc) on top of 1.5% isoflurane and remained significantly lower for 50 min after bolus administration [54]. Similarly, the first responses to peripheral electrical stimulation were detected only 60 min after administration of the initial medetomidine bolus (0.3 mg/kg ip), regardless of the CRI rate chosen for maintenance of anesthesia thereafter (0.1, 0.6 or 1.0 mg/kg/h) [41].

Findings reported in rsfMRI studies were more variable. Fc matrices between 92 components derived from independent component analysis (ICA), obtained 30 and 45 min after bolus (0.3 mg/kg sc, followed by 0.6 mg/kg/h sc), showed a significant difference only in 1.4% of connections, and graph theory-based analysis revealed no significant difference in the number of functional modules and small-worldness between the two timepoints [55]. Similarly, seed-based interhemispheric connectivity of six ROI did not significantly differ between 30 and 120 min after bolus administration within groups (0.3 mg/kg ip, followed by 0.1, 0.6 or 1.0 mg/kg/h ip), although thalamic interhemispheric fc significantly differed between 0.1 and 0.6 mg/kg/h only at the latter timepoint [46], indicating an interaction of dose and time effects. In contrast, the number of regions in the component most similar to default mode network as well as fc strength between selected regions increased from 20 to 50 min after a single bolus of medetomidine (0.3 mg/kg sc, [54]), and significant bi-directional differences in static fc were observed in several ICA-derived components between 20 and 55 min after bolus administration (0.05 mg/kg iv, followed by 0.1 mg/kg/h on top of 0.5% isoflurane, [56]). However, dynamic fc was minimally affected in the same study: The same set of “elementary building blocks (…) representing specific dynamic functional states” was identified at both timepoints, with significant increases in fluctuations at the latter timepoint observed only in 3/20 building blocks [56].

Taken together, the limited data suggests that while time-dependent effects occur under medetomidine alone or in combination with isoflurane, effects may be more evident in some regions and for some methods of analysis than others.

Summary

Overall, the included studies suggest that under medetomidine, alone or combined with a constant concentration of isoflurane, responses to peripheral stimulation as well as fc are suppressed in the initial phase after bolus administration [41,49,50,54]. Studies also consistently reported relatively stable measurements in the first 2 to 2.5 h of anesthesia across different types of BOLD fMRI [33,46,47,49,55]. The same tendency was observed in α-chloralose studies [50,51] but not consistently [47]. For medetomidine as well as α-chloralose, single reports found complex patterns of temporal evolution of responses to stimulation and fc when imaging was continued for more than 2 to 3 h [33,51]. 

This tendency for initial signal suppression, followed by a period of relatively stable measurements and complex patterns of signal evolution over time thereafter may however not apply to other injectable agents, and limited data was available for inhalant anesthetics. 

### 3.3. Comparisons between Specific Anesthetics

**Figure 3:**
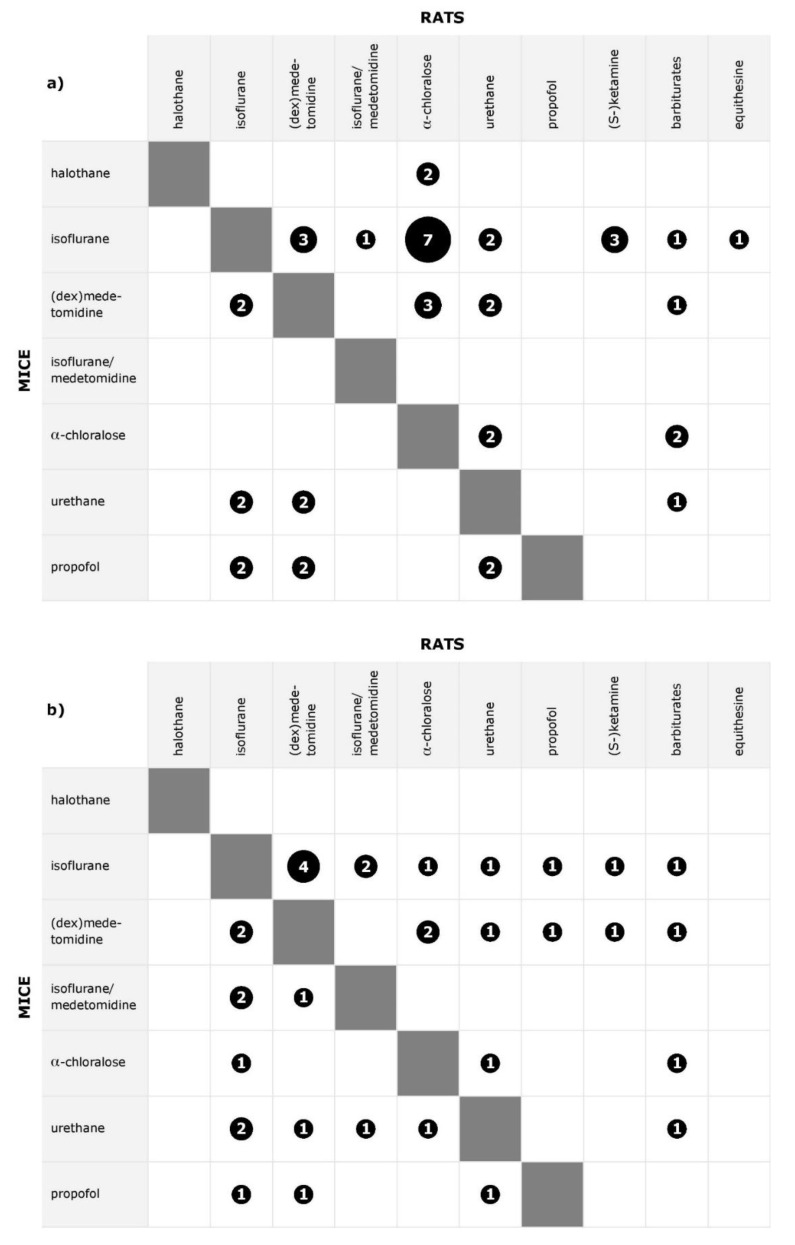
Distribution of pairwise comparisons of anesthetics for (**a**) stimulation fMRI and (**b**) rsfMRI. The size of the symbol represents the total number of datasets; the number of references may be higher.

An overview which anesthetics have been compared for stimulation and rsfMRI in rats and mice is given in Figure 3, and in more detail in Supplementary Materials Table S1, where for each reference it is listed which anesthetics were investigated. Studies which exclusively compared dose-dependence or time-dependence for two anesthetics are covered in previous sections, and results of studies which investigated effects of physiological parameter changes are covered in part a of this review [15]. Differences between anesthetics were found in all except three datasets (baseline BOLD signal after blood withdrawal, isoflurane versus halothane versus propofol [57]; response to peripheral electrical stimulation, medetomidine versus α-chloralose [50]; rsfMRI, isoflurane versus ketamine/xylazine [58]).

Among the studies directly comparing different anesthetic agents, only 4 out of 29 rat datasets compared more than two agents simultaneously, whereas 4 out of 6 mouse datasets compared multiple agents. As a result, no more than seven datasets were available per type of fMRI per specific anesthetic comparison. Additionally, outcome measures varied even within the same type of fMRI, so that reported findings were often complementary rather than allowing a conclusion for the consistency of findings. 

In the following section, findings for pairwise comparisons between α-chloralose, isoflurane, (dex)medetomidine and isoflurane/medetomidine are highlighted for both species, as those anesthetics were commonly investigated and are/were standard agents for BOLD fMRI studies. Additionally, comparisons of several anesthetics in mice are briefly summarized.

#### 3.3.1. Isoflurane versus α-Chloralose

Rats

With different modalities of peripheral and central stimulation, there was a trend for higher BOLD signal changes and spatially more extended activations under α-chloralose, but only one study compared α-chloralose and isoflurane for rsfMRI. 

Responses to peripheral electrical [59,60] as well as chemical [61] stimulation were of higher amplitude in at least some stimulation periods, and responses to peripheral mechanical stimulation additionally extended to more regions and activated more voxels within each region [62] under α-chloralose (40–80 mg/kg vi bolus, followed by 10–40 mg/kg/h iv, or 70 mg/kg ip) than isoflurane (1.0–2.0%). Responses to central electrical stimulation also showed higher amplitudes under α-chloralose (60 mg/kg iv, followed by 30 mg/kg/h iv) than isoflurane (1.0–1.25%) at some of the tested stimulation frequencies despite qualitatively similar frequency-dependence under both agents [63].

A study investigating responses to visceral stimulation furthermore noted partially agent-specific activation maps and that negative BOLD signal changes were more dominant under isoflurane (1.5%) than under α-chloralose (50 mg/kg/h iv) [64]. In contrast, no significant difference was observed in BOLD signal changes after nicotine administration in a single phMRI study [65]. When the same dataset was re-analyzed with a focus on fc, a correlation between nicotine-induced activation (area under the curve of the BOLD signal) and average coherence of ROI (measured across all ROI–ROI pairs) was however only found under isoflurane, and average coherence increased over time under α-chloralose (60 mg/kg iv, followed by 30 mg/kg iv every 60 min) as opposed to stable values under isoflurane (1.3%) [47].

Mice

A single resting state study in mice identified both similar and divergent components in ICA [66]. In seed-based analysis, differences were mainly observed when the seed was placed in S1: interhemispheric connectivity was significantly higher under isoflurane (1.0%) than α-chloralose (120 mg/kg ip), and clusters of voxels correlated to the seed had higher signal intensities under isoflurane, whereas cluster size and signal intensity was higher under α-chloralose when the seed was placed in caudate putamen. No differences were observed when the seed was placed in primary motor cortex (M1) [66].

Summary

Taken together, the included studies point towards tendentially stronger and/or more extended responses to stimulation under α-chloralose in rats, but insufficient data was available for rsfMRI and mice to identify agent-specific patterns.

#### 3.3.2. Medetomidine versus α-Chloralose

Rats

Only in rats medetomidine and α-chloralose were directly compared. While one study observed similar area of activation in S1 and similar peak signal changes in response to peripheral electrical stimulation under both anesthetics (medetomidine 0.05 mg/kg sc, followed by, 0.1 mg/kg/h sc, and α-chloralose 50 mg/kg iv, followed by 36 mg/kg iv every 60 min [50]), another study found that the stimulation frequency at which peak responses were obtained differed between medetomidine (0.1 mg/kg/h) and α-chloralose (46 mg/kg/h ip) [67], and accordingly, peak signal changes were significantly different at each agent’s optimal stimulation frequency. The same study also noted that responses were less reproducible within and between animals under medetomidine [67]. 

In a single phMRI study, the area under curve of BOLD signal changes after nicotine administration was significantly higher under medetomidine in cortical, but not subcortical regions [65]. When the same dataset was re-analyzed with a focus on fc, a correlation between nicotine-induced activation (area under the curve of the BOLD signal) and average coherence of ROI (measured across all ROI–ROI pairs) was only found under medetomidine, and average coherence increased over time under α-chloralose (60 mg/kg iv followed by 30 mg/kg iv every 60 min) as opposed to stable values under medetomidine (0.01 mg/kg iv, followed by 0.1 mg/kg/h iv [47]). Finally, the fractal index β of low frequency BOLD signal fluctuations was in cortical, but not subcortical regions higher under medetomidine (0.1 mg/kg/h ip) than under α-chloralose (40 mg/kg/h ip) [68].

Mice

No study directly compared medetomidine and α-chloralose in mice.

Summary

Insufficient data was available to identify agent specific patterns for different types of fMRI.

#### 3.3.3. Isoflurane versus (dex) Medetomidine

Rats

Isoflurane and (dex)medetomidine were not compared for peripheral, only for central stimulation in rats. Deep brain stimulation in the venteroposterior thalamus resulted in “extremely unstable” [69] responses under isoflurane (1.0–1.25%), as opposed to more reproducible localization of activation, activated area and signal amplitude under dexmedetomidine (0.025 mg/kg, followed by 0.05 mg/kg/h sc). Two phMRI studies found differences in responses to levo-tetrahydro-palmatine [70], but not nicotine [65]. Under isoflurane (1.4–1.6%), fewer regions were activated by levo-tetrahydro-palmatine, fewer regions showed negative BOLD signal changes than under medetomidine (0.1 mg/kg sc, followed by 0.1 mg/kg/h iv), and a significant effect of anesthetic agent on signal intensity plus interactions with the dose of the test-substance were found in multiple regions [70]. In contrast, similar regions were activated by nicotine, and area under the curve of BOLD signal responses obtained under isoflurane (1.3%) and medetomidine (0.01 mg/kg, followed by 0.1 mg/kg/h iv) differed neither in cortical nor subcortical regions [65]. Correlations between regional responses to nicotine and average coherence of ROI were found for subcortical regions under both agents, but for cortical regions only under isoflurane [47].

On a more general level, medetomidine was in rsfMRI associated with lower correlation strengths, but also more localized correlations [22,71,72,73]. Inter- and intrahemispheric connectivity values were generally higher under isoflurane than medetomidine ([73]; doses not reported), and average correlation values of voxels in contralateral S1 correlated to a seed in S1 were higher under isoflurane [71]—however, the ratio of correlated voxels in S1 to correlated voxels in the rest of the brain was lower under isoflurane, indicating less specific connectivity [71]. This and another study consistently reported widespread fc maps irrespective of the seed chosen under isoflurane (2% and 1.5%), whereas distinct fc maps with a focus on interhemispheric correlations between homotopic regions were obtained for each seed under medetomidine (0.05 mg/kg sc, followed by 0.1 mg/kg/h in both studies) [71,72].

Mice

Two studies comparing isoflurane (1.0%) and medetomidine (0.1 mg/kg iv, followed by 0.2 mg/kg/h iv) for peripheral electrical stimulation found agent-specific activation patterns, although activations were generally widespread and unspecific [38,74]. Under both agents, peak signal change did not differ between ipsi- and contralateral S1 and was linearly dependent on stimulus strength, and under both agents the size of activated clusters in somatosensory regions was similar [38]. Reproducibility of responses was however higher under isoflurane [38], and region-specific differences were observed in the exact signal time course of the BOLD response [38,74]. 

In rsfMRI, medetomidine (0.1 mg/kg iv, followed by 0.2 mg/kg/h iv) was, overall, associated with more pronounced connectivity in subcortical areas, whereas stronger cortical connectivity was reported under isoflurane (1%) [40,42,75]. Three publications analyzing the same dataset observed this trend for seed-based correlations [40] as well as ReHo [42] and connectivity within and between whole networks [75].

Summary

Taken together, no consistent pattern for responses to stimulation could be identified, but there was a tendency in rsfMRI for lower correlation values and spatially more localized fc under medetomidine in rats, and for stronger subcortical than cortical fc—relative to results obtained under isoflurane—under medetomidine in both species. 

#### 3.3.4. Isoflurane or Medetomidine versus Combined Isoflurane/Medetomidine

Rats

Addition of medetomidine (0.3 mg/kg/h) to previous monoanesthesia with isoflurane (i.e., without changing the isoflurane concentration of 1.3%) enhanced responses to peripheral electrical stimulation in terms of activated area in S1 and peak signal change in S1, and decreased interhemispheric connectivity for seeds in S1 and thalamus, but not caudate putamen [46]. When degrees of freedom of BOLD signal fluctuations were assessed as a measure of structured information content of the signal, values obtained under 1.5% isoflurane plus medetomidine (0.45 mg/kg sc, followed by 0.2 mg/kg/h iv) corresponded to those obtained under 2% isoflurane [25].

Medetomidine and isoflurane/medetomidine were not directly compared in rats.

Mice

Addition of a medetomidine bolus (0.3 mg/kg sc) to monoanesthesia with isoflurane (1.5%) decreased baseline signal intensity [54]. When medetomidine (0.05 mg/kg iv, followed by 0.1 mg/kg/h) was combined with a lower concentration of isoflurane (0.5%) than used in the isoflurane only condition, the combined protocol appeared to result in connectivity patterns which encompassed those observed under each anesthetic individually: Connectivity was strong in both cortical and subcortical areas, whether seed-based connectivity [40], ReHo [42] or whole networks were analyzed [75]. However, under isoflurane (1.3% or 1.5%) as well as isoflurane/medetomidine (0.5% plus 0.05 mg/kg iv, followed by 0.1 mg/kg/h) the same differences in fc maps between three mouse strains were evident [39].

Summary

While studies adding medetomidine to isoflurane without reducing the isoflurane concentration observed reductions in baseline signal, responses to stimulation and fc, the combination of medetomidine with a lower dose of isoflurane may enhance simultaneous detection of cortical and subcortical fc.

#### 3.3.5. Multiple Comparisons in Mice

In stimulation fMRI, two studies found agent-specific, but widespread bilateral responses to unilateral electrical paw stimulation under isoflurane, medetomidine, urethane and propofol [38,74].

rsfMRI tended to be characterized by agent-specific patterns of regionally enhanced or reduced fc strength. Cortical interhemispheric connectivity was lower under α-chloralose and urethane than under isoflurane in one study, and similar components were identified in ICA for more regions under α-chloralose and urethane than under isoflurane [66]. Another study found similar patterns of strong cortical and weak subcortical connectivity under isoflurane, urethane and propofol, strong subcortical and weak cortical connectivity under medetomidine, and strong cortical as well as subcortical connectivity under combined isoflurane/medetomidine [40]. When the same dataset was re-analyzed for ReHo, urethane, like medetomidine, showed in addition to high cortical ReHO also high ReHo in caudate putamen, and values in S1 were significantly lower than those obtained under isoflurane, indicating that depending on the outcome measure, fc patterns obtained under urethane may share similarities with those obtained under isoflurane or medetomidine [42].

### 3.4. Anesthetized versus Awake Imaging

**Figure 4:**
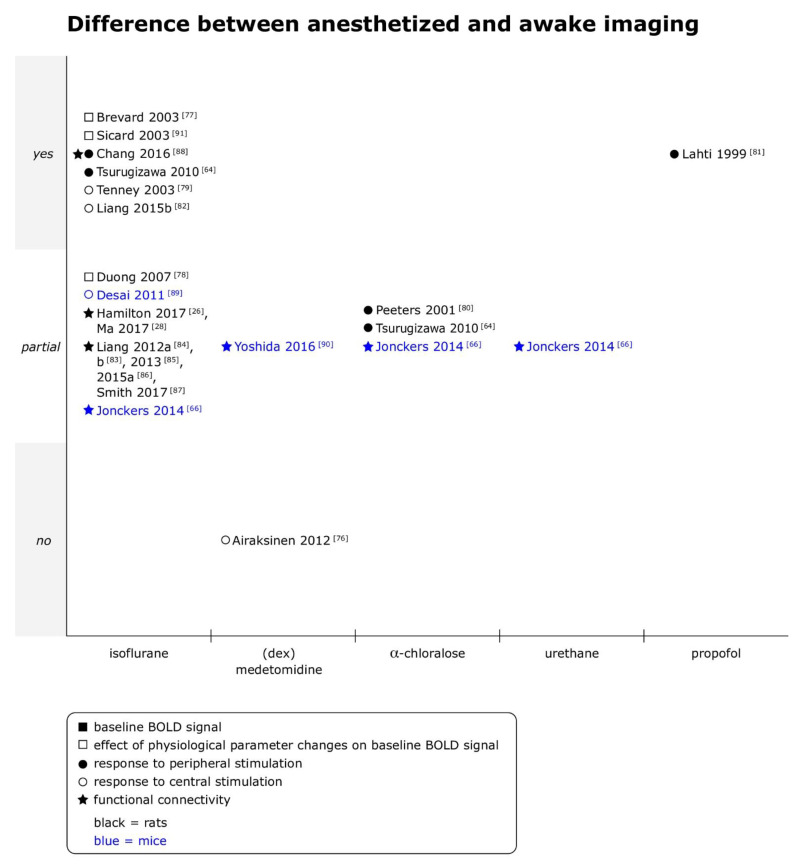
Comparison of anesthetized versus awake imaging for baseline BOLD signal, responses to peripheral and central stimulation and functional connectivity in rats and mice. Yes = differences between anesthetized and awake imaging were clearly observed; partial = differences between anesthetized and awake imaging were observed for some outcomes, analyses or animals, but not consistently, or quantitative differences were reported, but statistical significance unclear; and no = no differences between anesthetized and awake imaging were observed. When datasets were re-analyzed, findings were pooled and one data point per specific comparison (i.e., per anesthetic per type of fMRI) displayed. (dex)medetomidine = medetomidine or dexmedetomidine was studied. A summary of the main findings as well as the doses of anesthetics are provided for references with the symbols ■ or □ in Supplementary Materials Tables S2 and S5, with symbols ● or ○ in Supplementary Materials Tables S3 and S5, and for references with symbol ★ in Supplementary Materials Tables S4 and S6.

Anesthetized imaging was compared to awake imaging in 15 datasets for 19 combinations of anesthetic agent and type of fMRI (see Figure 4). Only in 1 out of 19 comparisons no difference was found in BOLD fMRI results between the two states [76]. Differences to imaging under isoflurane anesthesia were most frequently studied (12 comparisons), followed by differences to imaging under α-chloralose (3 comparisons) and medetomidine (2 comparisons).

Awake imaging studies typically fixed anesthetized animals on a cradle and started image acquisition once the animals had regained consciousness (11/15 datasets). An overview about the protocols for awake imaging is given in Table 2.

Rats

Stronger responses to stimulation in awake animals were commonly, but not consistently reported. Higher signal intensity and/or spatial extent of activations, potentially including unspecific areas, were observed across stimulation modalities—peripheral electrical stimulation [80,81], peripheral mechanical stimulation [88], optogenetic stimulation [82] and BOLD response to seizures [79], and in comparison to several anesthetics—α-chloralose (60 mg/kg bolus iv or 50 mg/kg/h iv), propofol (48 mg/kg/h iv) and isoflurane (1.0%–2.0%). In rats anesthetized with medetomidine (0.05 mg/kg sc, followed by 0.1 mg/kg/h sc) however, kainic-acid induced seizures elicited BOLD responses that did not differ from those in awake animals [76]. Additionally, intragastric administration of L-glutamate evoked negative BOLD signal changes in some regions in isoflurane- and α-chloralose-anesthetized, but not awake animals, and while isoflurane suppressed activations in expected regions, activations in additional regions were observed under α-chloralose [64]. 

Fc strength and/or extent was typically reduced in isoflurane anesthetized animals [26,28,83,84,85,86,87,88]. For example, studies reported less extended fc maps for seeds in in S1 [88], motor cortex [84] and thalamus [85], weaker connectivity between bilateral motor cortex [84] and more often reduced than enhanced fc within thalamocortical and frontoparietal networks [26] under isoflurane at concentrations ranging from 0.5% to 3%. One study noted that thalamocortical connectivity was significantly more reduced under isoflurane (2%) in associative than somatosensory networks [83]. However, two studies based on the same dataset also reported spatially homogenous reduction of averaged normalized fc of each voxel [26] and overall similar spatial patterns in ROI–ROI connections despite lower fc strength between ROI pairs under isoflurane (0.5–3.0%) [28]. In addition to those static fc measures, dynamic fc analysis approaches revealed differences in patterns between awake and isoflurane anesthetized animals. For example, clustering all connectivity matrices identified in sliding windows resulted in five patterns which all occurred in all scans, but specific patterns predominantly occurred in awake animals or at specific isoflurane concentrations [28]. Finally, one study analyzing global and local network parameters found no and minimal differences, respectively, between conditions, although regions were differently organized into modules when community structure was compared between isoflurane-anesthetized (2%) and awake animals [83].

Mice

In line with observations from stimulation studies in rats, a single study found that under isoflurane (0.7%), fewer regions were activated by optogenetic stimulation, and that peak signal change in S1 was significantly lower under isoflurane than in awake animals [89].

Although cortical and limbic networks were identified under medetomidine (0.3 mg/kg sc, followed by 0.6 mg/kg/h sc) as well as in awake animals, connectivity between specific regions, notably with somatosensory cortex, was reduced or absent under medetomidine [90]. Another study comparing awake imaging to imaging under isoflurane (1.0%), α-chloralose (120 mg/kg ip) and urethane (2.5 g/kg ip) found overall similar, spatially “not completely overlapping” components in ICA in several regions, but also identified components in caudate putamen which were present in all anesthetized groups as opposed to awake animals [66]. Seed-based analysis revealed larger cluster size of voxels correlated to seeds in S1 in awake animals, and higher peak signal intensity in clusters correlated to seeds in S1 and M1 in awake animals. Interhemispheric connectivity between S1 was also significantly stronger in awake animals [66].

Summary

Taken together, responses to various types of stimulation were generally weaker and/or spatially less extended in anesthetized animals in both species. Functional connectivity was consistently reported to be reduced in strength and/or extent, or to express different dynamic patterns in anesthetized compared to awake animals, although individual studies found regional differences in susceptibility to those effects (see Supplementary Materials Tables S4 and S6 for more details).

## 4. Discussion

The present systematic review analyzed the effects of different anesthesia protocols on BOLD fMRI readouts in mice and rats. Studies included compared the same agent at different doses or different time points following their administration, or investigated certain agents relative to other agents or the awake state. 

In the following, the findings are discussed with a focus on isoflurane and (dex)medetomidine, and practical implications are derived for anesthetic protocol design for BOLD fMRI in general.

### 4.1. Dose Effects

Anesthetic doses commonly showed an effect on fMRI results, but reported effects were not necessarily monotonic. Dose-dependence of results was more pronounced under isoflurane than (dex)medetomidine, and under (dex)medetomidine, rsfMRI was more affected than stimulation fMRI. This dose-dependent effect is a combined consequence of agent-specific influence on cardiovascular and neural function. 

Overall, with deeper levels of anesthesia, as shown with EEG (electroencephalogram) and local electrophysiological recordings [11], neural activity (spontaneous and evoked) is reduced. The summation of evoked field potentials [92] as well as somatosensory evoked potentials are reduced with increasing concentrations of isoflurane, and somatosensory evoked potentials are almost absent at 3% isoflurane [22]. Around 1.8% to 2% isoflurane burst suppression occurs [22,27,92], whereas at 3% minimal EEG power across different frequencies is recorded [22].

Cardiovascular effects caused by isoflurane are primarily the result of dose-dependent vasodilation. Dilation of systemic vessels on one hand reduces mean arterial blood pressure (MAP), whereas dilation of cerebral vessels may increase CBF [93,94,95], although changes in baseline CBF are inconsistently found [92]. Baseline BOLD signal intensity was increased with higher isoflurane concentrations, probably as a combined consequence of reduced O_2_ consumption, due to lower metabolism and O_2_ extraction, and increased CBF [19,94]. Reduced responses to stimulation under isoflurane anesthesia might be explained by two mechanisms: On one hand, dilation in response to stimulation might be supramaximal in vessels that are already dilated at baseline [94], and increases in local blood flow and oxygenated hemoglobin are limited [9]. On the other hand, there is evidence that isoflurane directly interferes with neurovascular coupling [92]. What needs to be considered in this context is that FP, CBF and BOLD responses are also stimulation frequency-dependent [96], and that dose-dependent trends cannot be generalized for all stimulation frequencies [92].

At isoflurane concentrations just above the minimal alveolar concentration (MAC), more extended fc maps and stronger interhemispheric connectivity occurred probably due to lower MAP at higher concentrations. In mice its known that MAP is significantly lower at 2% isoflurane compared to 1% [95] and that a decrease of MAP from 110 +/- 10 to 68 +/- 7 mmHg increases CBF fluctuations and spontaneous BOLD signal fluctuations [97]. 

Non-monotonic trends noted for some rsfMRI outcomes and suspected inverse U-shape might be a consequence of several mechanisms. Either the effect of increased CBF be offset by reduced neural activity at certain concentrations, or maximal isoflurane concentrations reduce MAP beyond autoregulatory limits, with the consequence that CBF cannot be maintained, and/or interference of isoflurane with neurovascular coupling affects the generation of underlying low frequency BOLD signal fluctuations.

The less pronounced dose effects under (dex)medetomidine compared to isoflurane can be explained by less dose-dependent neural and cardiovascular effects of (dex)medetomidine. When infusion rates of 0.1 and 0.3 mg/kg/h were compared [22] somatosensory evoked potentials were identical. Generally (dex)medetomidine EEG pattern resembles non-rapid eye movement (NREM) sleep [98]. With higher medetomidine plasma concentrations EEG frequencies decrease, and amplitudes increase [99], but when in rats the dose of a single bolus is increased above a certain threshold, only duration of sedation, but not depth of sedation is increased [100]. (Dex)medetomidine contrary to isoflurane is a potent vasoconstrictor [101], and effects on cerebral vasculature do not depend on the dose administered [94]. As expected, BOLD responses to stimulation, for the most part of the included studies, were not influenced by the dose of (dex)medetomidine. In all rsfMRI studies however also (dex)medetomidine showed dose dependent effects, that were weaker in caudate putamen, where α2-receptor density is reduced in comparison to the cortex [102]. As cerebral hemodynamics are less influenced by variable doses of (dex)medetomidine than by variable concentrations of isoflurane, dose-dependence recorded in rsfMRI probably primarily is a consequence of altered neural activity.

However, (dex)medetomidine may not be the single agent under which responses to stimulation are less sensitive to anesthetic depth than rsfMRI measures: Electrophysiological recordings and rsfMRI studies have shown that sensory networks are less sensitive than association networks to anesthetic depth [86,103].

### 4.2. Time Effects 

Not only the dose of an anesthetic, but also the time point of imaging relative to induction and/or eventual boli will affect the observed outcomes, and interactions between dose and time effects may be hard to separate. Following a bolus of (dex)medetomidine or α-chloralose, fc and response to stimulation were suppressed [41,49,50,54], followed by a phase of relatively stable measurements within 2 to 2.5 h after start of administration [41,47,49], and complex patterns of temporal evolution thereafter [33,51]. In contrast, no time-dependent effects were reported during exposure to isoflurane, but the duration of isoflurane exposure during preparation affected results obtained later under a dexmedetomidine CRI [48].

Two key factors must be considered concerning time dependence of fMRI results. First, it is difficult to reach a steady state, defined as constant agent concentrations in the volume of distribution [104], and thereby constant drug concentration at the effect site, with injectable anesthetic agents. Second, as anesthesia affects a dynamic system, even with constant drug concentrations adaptions of neurons and networks might occur over time and such changes might even outlast the duration of exposure. 

When a single bolus is administered, plasma and effect site concentrations (i.e., in the brain) reach a peak before declining again. As a consequence, anesthesia is deepest within a certain time after the bolus and gets lighter thereafter. The exact time course depends on several factors such as route of administration, distribution and metabolism of the agent [105], and varies between individuals. Such variable anesthetic plasma and target organ concentrations will result in neural and cardiovascular effects that influence BOLD signal. 

For medetomidine for example it is known, that in rats within 10 min after sc administration of a bolus (80 μg/kg) peak plasma concentrations are reached. Maximal concentrations in the brain are reached within 15–20 min after bolus administration [106]. Studies administering a medetomidine bolus prior to starting a CRI consistently reported that responses to stimulation were detected only from 60 to 80 min after an ip [41] or sc [50] bolus on, which likely mirrors the time-course of concentrations at effect site; likewise, stronger and more extended fc was reported 50 than 20 min after a single bolus of medetomidine not followed by a CRI [54]. After the initial bolus, a CRI is usually started, but no data on plasma or effect site levels following constant rate infusions are available for rodents. It is important to note that constant rate administration of anesthetics does not result in constant concentrations in the brain [107], and clinical observations suggest that—unlike in other species [108,109]—constant levels of sedation are not achieved with prolonged medetomidine infusions [33,110]. Additionally, seizure-like activity evoked by electrical paw stimulation has been reported from 2h after start of a dexmedetomidine CRI on [111].

Depending on the time span covered, the included studies reported no [47,55] or minor [41,48] differences in fc over time under (dex)medetomidine for timepoints which were 15 to 60 min and 2 to 6 h, respectively, apart. In contrast, correlation coefficients significantly decreased over time when the initial bolus was omitted [33]. This last observation is in line with findings of a more recent publication (i.e., published after the systematic search was performed and therefore not included in the main analysis) where in the first 2 h of medetomidine administration mean correlation coefficient across all ROI pairs was significantly higher than in later phases when no bolus was given, and also significantly higher during that early phase than in protocols with an initial bolus [110]. Four different medetomidine administration protocols (iv administration of 0.05 mg/kg followed by a CRI of 0.1 mg/kg/h, versus sc administration of the same protocol, versus iv administration of half the doses, versus iv administration of just the CRI of 0.1 mg/kg/h) and temporal stability of responses to paw stimulation and fc were compared in that study. While localization of the center of activation and the hierarchical structure of networks were stable over time under all protocols, a significant effect of time and time-protocol interaction on peak signal change, and a significant effect of time, protocol and time-protocol interaction on mean correlation across all ROI pairs was detected. Peak signal changes significantly decreased from the first 2 h to later phases under the sc, half dose and CRI-only protocols. Taken together those observations suggest that stable medetomidine levels were reached only after 2 h when no bolus was administered, the dose reduced or the sc route chosen. This interpretation can however not explain why in the above cited study [33] increasing the rate after 2 h prevented the decrease in correlation coefficients observed with a constant rate. Overall, dose and time effects are tightly connected, depend also on the route of administration, and are currently insufficiently characterized for (dex)medetomidine. 

Although α-chloralose has been used for several decades in rodents, even less is known about its pharmacokinetics. Studies report that α-chloralose will produce superficial anesthesia for 8–10 h [107]. Protocols giving top-up boli at a certain interval and protocols starting a CRI after the initial bolus were represented among the included studies. As repeated bolus administration generally results in fluctuating agent concentrations [105], it is not surprising that two studies using top-up boli reported changes in responses to central stimulation [51] and in fc over time [47]. To the best of our knowledge, no plasma concentrations for any of the protocols in use have been measured (see Supplementary Tables S2–S6 for α-chloralose administration protocols included in this review).

Another aspect to consider with injectable anesthetics is that tolerance might develop with prolonged or repeated exposure. Possible mechanisms are reduced neuronal sensitivity or increased drug metabolism [112]. For example, in rats with repeated ketamine use, induction of hepatic enzymes occurs and anesthesia time following identical dose decreases [113,114]. With propofol in rats within 90 min of its infusion, signs of tolerance were noted in EEG measurements [115]. In our review no study investigated whether ketamine or propofol induced tolerance. Only for medetomidine there is some data investigating constant drug administration and its effects. In rats it was observed that despite a constant infusion of medetomidine (0.1 or 0.3 mg/kg/h iv), and independent of the dose rate tested, they woke up after 3.5 to 4 h of infusion [33]. When the rate of infusion 2.5 h after bolus administration was increased, the duration of sedation was prolonged to 6 h, so probably within the studied timeframe no tolerance occurred. A recent publication found that 73% of anesthetic sessions with four different medetomidine administration protocols in rats lasted more than 5 h, but only in 56% of sessions animals remained anesthetized for the full 6 h study period [110]. Those wake-up events occurred with all administration protocols (sc and iv, with and without initial bolus) and in bench-top as well as fMRI sessions. Unfortunately, neither study allowed differentiation between pharmacokinetic and -dynamic mechanisms because plasma concentrations were not measured. In mice, maximal duration of sedation achieved with medetomidine appears to be even shorter, only around 2 h [116].

With modern inhalant anesthetics (isoflurane, sevoflurane) on the other hand, drug concentrations at effect site are easily maintained within a narrow therapeutic window. Partial pressure of the anesthetic in the brain is in line with its partial pressure in the alveolus, once the patient is in a steady state anesthesia (which is the case within minutes in small rodents). End-tidal continuous monitoring of inhalant concentration can be performed in intubated animals as a surrogate of inhalant anesthetic concentration in the brain. In usually for rodents used non-rebreathing anesthesia systems, the inhalant delivery is with high fresh gas flows, allowing rapid changes in partial pressures of the inhalant and depths of anesthesia [105]. As a consequence, in comparison to injection anesthesia, inhalation anesthesia is easy to guide and easy to monitor. Steady state anesthesia is readily achieved with isoflurane or sevoflurane and tolerance does not occur [117]. Nevertheless, even at constant levels of anesthesia changes in the dynamic system of the brain might occur. Such changes eventually remain even beyond the anesthetic itself. One study reported that following isoflurane exposure, rsfMRI outcomes under dexmedetomidine CRI were affected for a time period exceeding the washout period of isoflurane [48]. Further studies are warranted to analyze this effect in more detail. 

In summary, based on detailed pharmacodynamic and -kinetic data for the target species and the anesthetic(s) of choice, optimal time spans for image acquisition should be identified. Ideally anesthesia should during the whole imaging period be at steady state. The (possibly negative) role of isoflurane, that is used in many studies for anesthesia induction and preparation before switching to another anesthesia regime, needs to be further analyzed and characterized.

### 4.3. Comparisons between Specific Anesthetics 

The vast majority of included studies (33/36 datasets) did find differences in BOLD fMRI results when anesthetic agents were directly compared. Due to the diversity of anesthetics, types of fMRI and outcome measures, trends for agent specific patterns could however only be identified for a minority of subsets.

For decades α-chloralose, as it preserves functional-metabolic coupling [118], was the standard anesthetic in neuroscience and it was often used in the first 15 years of BOLD fMRI. In stimulation studies of this review α-chloralose is overrepresented as rsfMRI of mice and rats became only in the mid-2000s popular. In rats a trend for higher BOLD signal changes and/or spatially more extended activations was noted with α-chloralose across all stimulation paradigms in comparison to isoflurane. However, α-chloralose leads to excitatory and long recoveries [107] and causes inflammation when administered ip [119] and should not be used in recovery studies. Moreover, it should not be used for anesthesia induction, as onset of action is too slow resulting in excitations during this phase [107,120]. As α-chloralose is not a registered medical product, solutions have to be prepared in-house for each experiment, resulting in a product of unknown quality [120]. Although responses to stimulations measured were robust, α-chloralose is not used often anymore for BOLD fMRI.

Data about effects on fc was sparse among the included studies, with one study finding more independent components similar to those identified in awake animals under α-chloralose than under isoflurane and weaker connectivity than under isoflurane [66]. This observation is supported by a recent study describing globally suppressed, but qualitatively preserved cortical fc patterns under α-chloralose compared to awake animals [121]. However, the combination of isoflurane and medetomidine also resulted in “moderate to good” representation of fc patterns found in awake animals, and fc patterns were even better preserved under propofol. Given its significant drawbacks, α-chloralose would need to provide substantially more representative and reproducible results than other agent(s) to justify its broad use. 

As BOLD fMRI started later in mice than in rats, studies in this species from beginning on used rather isoflurane and (dex)medetomidine than α-chloralose. In stimulation studies in rats, isoflurane and (dex)medetomidine yielded satisfactory results but were only rarely directly compared (one deep brain stimulation [63] and one phMRI study [65]). In mice, isoflurane and medetomidine were compared for peripheral electrical stimulation studies. Despite unilateral stimulation, bilateral signal changes were reported with both anesthetics in those studies which directly compared the two agents, and heart rate, pulse distension and SpO_2_ changed upon stimulation [38,74]. As bilateral responses were not expected based on fMRI studies in other species and electrophysiological reports, they were interpreted as an artefact due to cardiovascular arousal. In the same two studies, cardiovascular reactions to stimulation and unspecific bilateral signal changes were also observed under urethane and propofol anesthesia, suggesting this is a general challenge in mouse stimulation fMRI [38,74]. However, other studies which investigated just medetomidine reported unilateral responses to electrical paw stimulation [41,116]. Whether specific responses are obtained under medetomidine may depend on stimulation parameters as well as doses used: The studies finding unilateral responses used higher absolute doses (0.3 mg/kg sc, followed by 0.6 mg/kg/sc [116] and 0.3 mg/kg ip, followed by 0.1, 0.6 or 1.0 mg/kg/h ip [41], as opposed to 0.1 mg/kg iv, followed by 0.2 mg/kg/h iv [38,74]), and with the lowest dose in the second study, 3 of 11 animals were excluded due to widespread responses to stimulation interpreted by the authors as indicative of “pain” [41], although the different routes of administration make it difficult to directly compare the doses.

Unilateral responses to electrical paw stimulation were recently obtained under a ketamine/xylazine protocol, but not under isoflurane [122]. Under ketamine/xylazine the mice were markedly bradycardic throughout the study and heart rate did not increase upon stimulation. Therefore, the findings might be explained by a suppressed cardiovascular response. Mechanistically, rapid changes in blood pressure or cardiac output may override cerebral autoregulation and result in additional blood flow to regions without underlying neuronal activation (for a more detailed discussion, see [15]); and indeed, regional changes in BOLD signal upon stimulation were correlated with estimated O_2_ delivery (based on changes in CBV and SpO_2_ at the time of stimulation) in a previous study [38]. Suppressing the cardiovascular response to stimulation is consequently expected to reduce unspecific signal changes. α2-agonists, such as xylazine and (dex)medetomidine, cause marked bradycardia [123]. However, increases in heart rate upon stimulation were observed under medetomidine alone [38], indicating that ketamine’s analgesic effect [124] might have blunted nociceptive contributions to the cardiovascular response in the combined ketamine/xylazine protocol. Only further studies will enable us to confirm the results and to establish ideal doses and time courses for optimal imaging. Which α2-agonist is optimal for combination with ketamine, it might also need some further investigation. Ketamine/dexmedetomidine administered ip has the advantage of causing less local irritation than ketamine/xylazine, but at comparable levels of anesthesia it causes more cardiorespiratory depression [125]. 

The combination of isoflurane/dexmedetomidine was not tested in the included stimulation studies in mice. A recent publication found that adding 0.3% of isoflurane to a dexmedetomidine CRI (0.4 mg/kg sc, followed by 0.8 mg/kg/h) reduced both spatial extent and strength of responses to olfactory stimulation [126]. In rats, the combination of medetomidine and 1.3% isoflurane resulted in larger activated areas and higher signal amplitudes than those observed under isoflurane alone upon electrical paw stimulation [46]. Additionally, the seizure-like activities in response to electrical paw stimulation detected in EEG recordings under medetomidine were prevented when 0.3% isoflurane was added [111]. To assess the usefulness of (dex)medetomidine and isoflurane combinations for stimulation fMRI and to identify optimal doses of each drug, more studies are warranted. For rsfMRI, medetomidine combined with low dose isoflurane yielded promising results. Only their combination resulted in strong cortical and subcortical fc in mice [40,42,75], whilst medetomidine alone was associated with stronger subcortical fc and isoflurane with stronger cortical fc. These findings were recently reproduced in rats, where widespread network structures and enhanced fc in cortical and striatal regions were observed under isoflurane, as opposed to a global suppression of fc with particularly strong effects on cortical connectivity under medetomidine [121], and support the trend for more specific, but weaker fc under (dex)medetomidine that was evident in the included rat studies.

Overall, the available data was too heterogenous to identify robust patterns of agent specific effects on specific types of fMRI and outcome measures, and open questions remain about optimal protocols for specific types of fMRI.

### 4.4. Anesthetized versus Awake Imaging

In awake animals, compared to animals anesthetized with isoflurane, medetomidine, propofol, α-chloralose or urethane, responses to stimulation trended to be of higher signal intensity and/or spatial extent [76,77,78,79,88,89] and fc of higher connectivity strength and/or spatial extent [26,28,66,80,81,82,83,84,85,90].

Whether the higher signal intensity and larger activated areas in awake animals were interpreted as desirable varied between different types of stimulation and anesthetics; less specific [81] or reproducible [80] responses to electrical paw stimulation were reported under propofol and α-chloralose, respectively, but enhanced detection of responses to mechanical paw stimulation [88] and optogenetic stimulation [82,89] compared to those obtained under isoflurane. A recent publication in mice even reported activations extending to regions not involved in visual processing under ketamine/xylazine anesthesia, while activations were limited to expected regions in awake animals upon visual stimulation [127]. Taken together, those observations suggest that whether more specific responses are obtained in awake or anesthetized animals may depend on the type of stimulation as well as on the selected anesthetic. 

Fc in awake rats and mice was in all studies more pronounced in at least some aspects. While studies included in this review compared at maximum three anesthetic protocols to awake imaging, a recent publication systematically investigated effects of six anesthetic protocols on fc matrices, region-specific mean correlation coefficients, fc within the default-mode network, complex network parameters and spectral distribution of BOLD signal fluctuations in rats [121]. Fc patterns were most similar to those found in awake animals under propofol or urethane, and least similar under isoflurane or medetomidine, with intermediate similarity under combined isoflurane/medetomidine or α-chloralose. In contrast to findings of this review, cross-correlation values were generally enhanced under isoflurane. Interestingly, the combination of isoflurane and medetomidine appeared to “balance out” agent specific effects (suppression of fc under medetomidine/enhancement under isoflurane), resulting in a “moderate to good” correspondence to findings in awake animals. 

However, although commonly presented as a gold standard, fMRI measurements in awake rodents may not, per se, provide the most accurate data. In human fMRI it is well recognized that spontaneous cognition may introduce additional, unspecific activation or connectivity [128]. Some of the included studies in this review reported unspecific connectivity or responses to stimulation in awake animals which is not surprising as restraint and noise are known stressors to rodents. The fact that physiological parameters within normal ranges for awake animals were reported during scanning [77] does not implicate that those animals were not stressed, as the same authors noted that struggling movements “markedly decreased after the first hour”. Options for monitoring physiological parameters are generally more limited in awake animals, which may not tolerate equipment such as pulseoxymetres, resulting in inaccurate readings and additional stress. It is therefore more challenging to assess baseline physiological status in awake animals and detect in particular cardiovascular responses to stimulation than in anesthetized animals. 

To reduce stress of awake imaging acclimation protocols have been proposed, in which animals are briefly anesthetized for fixation on the cradle and then kept restrained for increasing durations. King et al. [129] reported that in rats from day three of training on respiratory rates were lower and heart rates and corticosterone levels from day five on. Another study in rats found decreases in respiratory and heart rates on days four and five of training [64]. Contrary to this, mice showed consistently higher respiratory rates during scanning compared to freely moving mice and it was concluded that repetition of exposure did not decrease stress in mice [66]. A recent study found that corticosterone levels were elevated on day 5 of training and decreased to the high-normal range, but above pre-training values, on day 10 of training [127]. Interestingly, the same study also noted that motion during the actual imaging session was reduced if the last 3 of 10 acclimation sessions took place in the actual scanner instead of a mock scanner [127].

Acclimation protocols might reduce motion artefacts, but the repeated stress those protocols cause may on its own have an influence on BOLD fMRI outcomes. In rats it has been shown that daily immobilization for 2 h during 10 days resulted in increased connectivity in “somatosensory, visual and default mode networks” [130]. On the other hand, rats undergoing an acclimation protocol used in several of the included studies did not show more anxiety on an elevated plus maze 12 days after the last acclimation session [131]. Initial inhalation anesthesia for positioning the animals is a perseverative source of aversion and stress to the animals, as it is known that anesthesia, per se, leads to a stress response [132] and isoflurane and sevoflurane induce aversion with repeated exposure [133,134,135,136]. 

Furthermore, effects of a short anesthesia for positioning might extend into the period of imaging: In humans it was reported that cognitive failure occurred up to 3 days after regaining consciousness [137], and in rats, effects of isoflurane on fc persist beyond its residual presence in the body [48,59].

Three of the included studies therefore either just omitted anesthesia for positioning of the animals or trained animals to voluntarily tolerate fixation. With repeated restraint, stress markers were reduced from day six of training on, whilst muscle activity was already reduced on day one [90]. Training to tolerate fixation in rats resulted in corticosteroid levels not different from baseline and respiratory rates lower than with other awake imaging strategies, equal to anesthetized animals [88,129]. Nevertheless, acclimation was not able to completely avoid stress of the actual imaging session, as right following awake imaging corticosterone levels were still higher than at baseline or during training.

To reduce motion artefacts, two studies used neuromuscular blockade after the end of inhalant anesthesia [76,80], which must have caused awareness during muscle paralysis known to be unacceptably stressful in humans [138,139], and thus also in animals this practice seems ethically not justifiable.

In summary, awake imaging is not “confounder-free”. Stress associated with the actual imaging session, as well as the potential of acclimation protocols to reduce it, require further investigation including a broader range of indicators than physiological parameters, for example behavioral testing right after exposure. Knowing the degree of stress associated with awake imaging is relevant for animal welfare assessments as well as for the validity of results. Training animals to voluntarily tolerate awake imaging may be a promising, yet labor-intensive approach for stress reduction.

### 4.5. Practical Implications 

Our results have shown that anesthesia most likely will affect BOLD fMRI outcomes. However, as awake imaging is not “confounder-free” either and not feasible when fMRI is combined with more invasive techniques, imaging animals under anesthesia remains a legitimate option. 

Once the decision has been taken to perform a BOLD fMRI experiment under anesthesia, an anesthetic protocol with suitable drugs and doses has to be chosen and practical aspects and timing have to be included. Anesthetic agents are suitable if BOLD results correspond with directly measured neural activity and if they provide stable conditions throughout data acquisition. Although the low number of mouse studies included in this review did not allow to identify consistent species differences in the way anesthetic protocols affect BOLD fMRI results, optimal protocols for rats and mice may differ: Minimum alveolar concentrations of inhalant anesthetic are slightly higher in rats [140], metabolic rate is higher in mice (which may impact pharmacokinetics of injectable agents) and species differences in cerebral autoregulation have been reported [141]. While conclusions presented here apply to both species, protocols should be optimized for rats and mice separately, taking also into account strain differences within each species.

A first, practical consideration are the requirements for anesthesia induction and preparation versus imaging. Not all agents are suitable for anesthesia induction (α-chloralose for example is not), and invasive procedures prior to imaging may require a deeper plane of anesthesia than later during imaging, and/or additional analgesia. Most often isoflurane, as it is simple to use and wears off quickly, is used initially before maintenance of anesthesia is switched to injectable anesthesia. Carry-over effects may however result if washout times of inhalants are not awaited. If invasive procedures are planned during preparation, adequate analgesia needs to be included in the anesthetic protocol, notably when anesthesia is maintained with isoflurane. Fluid management is another important aspect of anesthetic protocols, especially for longer procedures or when α2-agonists are used, which cause marked diuresis [142,143]. Care must be taken that all experimental groups receive identical fluid doses, and volumes of drugs and catheter flush solutions should not be neglected.

Parasympatholytics (atropine, glycopyrrolate) have a strong influence on cardiovascular function and fading concentrations during imaging will have unpredictable effects on the hemodynamic basis of the BOLD signal; thus, their use should be omitted. Together with α2-agonists they can even lead to fatalities as protective reflex-bradycardia is suppressed [123]. 

The selection of anesthetic agent(s) for the imaging part of the experiment must be guided by evidence gained in comparison studies. In case of phMRI, anesthetic agents with possible interactions with the tested drugs have to be avoided.

The anesthesia for imaging should provide stable anesthesia during the whole period of data acquisition: Not only should agent concentrations be as close to steady state as possible, but also physiological parameters should be stable and alternative measurements of brain activity (e.g., from electrophysiologic or optical methods) should provide evidence for unchanged activity over the relevant timespan. Additionally, such regimen should be suitable for longitudinal (recovery) studies. 

A steady state of anesthesia is ideally maintained throughout imaging. It is easier to reach and maintain steady state anesthesia with inhalant anesthesia, in particular for prolonged periods of time. Studies in this review typically used isoflurane. However, isoflurane may not be the ideal agent. It has strong vasodilatory effects resulting in slower and weaker hemodynamic responses to stimulation [144]. Moreover, unspecific bilateral BOLD signal changes in unilateral paw stimulation studies in mice have been reported [38,122], as well as pronounced differences of fc patterns under isoflurane to those found in awake rats [121]. 

Sevoflurane, that might be a better alternative as it maintains cerebral autoregulation better and causes less increase of CBF than isoflurane [93,145,146], has not yet been investigated in the included studies but is certainly worth further investigation. Injectable agents on the other hand have more complex pharmacokinetics, and it is therefore essential to know or determine the time window during which anesthesia approaches steady state when validation studies suggest that a specific injectable provides promising results [121,122]. Overall, suitable anesthetic protocols need to find a balance between anesthetic stability and permitting representative measurements of the functions, pathways and networks under investigation.

Dose rates have a major influence whether valid measurements can be acquired under a specific agent. In one study, included in this review, that compared four different anesthetics a dose reduction of each drug resulted in less differences in fc patterns [40]. The goal for most studies is therefore to reduce anesthetic doses to a point where agent specific neural depression and cardiovascular side effects are minimized, but arousal and cardiovascular instability prevented [38,95]. Even lower doses of individual agents can be used when different agents are combined to exploit synergistic effects. This concept of balanced anesthesia is well established and widely applied in clinical anesthesia [147], and its application in rodent BOLD fMRI may yield data which is more representative of generally applicable mechanisms, as agent-specific influences are reduced. For instance, combining medetomidine with low doses of isoflurane preserved cortical and subcortical connectivity, in contrast to monoanesthesia with either agent that showed strong influences on either cortical or subcortical connectivity [40,121]. However, further studies are needed to determine ideal dose combinations, characterize temporal stability and explore other agent combinations (e.g., sevoflurane and (dex)medetomidine). For each specific combination time, windows with stable anesthesia conditions have to be defined, as the more agents are involved, the more pharmacokinetic and pharmacodynamic interactions have to be expected. During all further dose-finding studies it is important that imaging conditions are exactly imitated in bench-top experiments, particularly the strong stimulation from scanner noise has to be accounted for.

BOLD fMRI studies should standardize the timing for each step of the experiment and protocol it individually, as timing of image acquisition was shown to affect BOLD readouts in most included studies. If a different route of administration is chosen than described in the literature, the window of stable anesthetic conditions may deviate from published findings for that agent, because uptake and time courses of plasma and effect site concentrations may vary between routes of administration; for example, temporal stability of measurements differs between sc and iv administration of medetomidine [110]. When animals of variable size are investigated, dosing per bodyweight rather than “per animal” (i.e., administering a fixed total dose) prevents unintended variation in individual doses. Strain and sex differences that have been reported in in rats [148,149,150,151] as well as in mice [39,136] should be considered. Adjustments to published doses should be based on literature or better on pilot studies. 

In conclusion, for each species standard protocols, tailored to specific experimental conditions, should be established. Suitable, well designed balanced anesthetic protocols tested in pilot studies in combination with careful monitoring (see part a of this review [15]), form the basis of interpretable, reproducible and comparable fMRI results.

### 4.6. Limitations 

Concerning the conduction of the present review, the facts that a single reviewer performed study selection and that only a quarter of data was extracted by two reviewers are limitations. If references did not clearly meet the in- or exclusion criteria, the decision was discussed with the supervisor (R.B-W.) and the decision documented. However, this approach does not provide the degree of objectivity that would be achieved by two completely independent reviewers. 

Concerning the included references, a major limitation is that all publications included were judged having a high risk of bias, which means that the strength of evidence of this review is weak and future research might complement or even correct our current results [152]. 

As a result of the broad inclusion criteria, different types of BOLD fMRI experiments and various outcome measures were represented among the included studies, so that despite the large total number of studies only few reports were available for any specific comparison. 

Finally, instable physiological parameters or differences in physiological parameters between experimental conditions may have confounded some of the reported observations. For example, a study investigating responses to paw stimulation over time under dexmedetomidine CRI [49], recorded over the observation period a decrease in arterial partial pressure of CO_2_ from 62 to 50 mmHg, which on its own will change responses to stimulation. However, as evidence for effects of physiological parameters was collected in parallel to the analysis of differences between anesthetic protocols, we did not assess study quality based on reported physiological parameters. 

## 5. Conclusions

The present systematic review has shown that anesthetic agents, their doses as well as time points of imaging affect BOLD signal across different types of BOLD fMRI, and that anesthetic protocols therefore might confound BOLD fMRI measurements. The variety of currently used protocols should be narrowed down to a selection of standardized protocols with known impact on outcome measures, and particularly the contribution of physiological parameters requires further investigation. Standardization of doses, routes and timing of administration warrants stable levels of anesthesia within defined time window. Establishing a selection of standardized anesthetic protocols, which also include adequate monitoring and management, will increase the reproducibility of results, facilitate comparison of results between studies and enable synthesis in meta-analysis, and thereby improve the scientific validity of BOLD fMRI studies in rats and mice. 

## Figures and Tables

**Table 1:** Inclusion and exclusion criteria.

Category	Inclusion Criteria	Exclusion Criteria
Type of study	original research short form (conference abstract, poster or paper) or full article re-analysis of previously acquired data	review opinion piece book chapters lecture/talk study protocol short form with corresponding full article multiple reporting unrecognized duplicate abstract collections or conference proceedings retrieved as one reference
Type of animals	rats >200 g or 8 weeks and up to 12 months ^1^ mice >18 g or 8 weeks and up to 12 months ^1^ if age/weight not reported: Assumed that animals were adult unless stated otherwise both sexes any strain any health status	species other than rat or mouse rats <200 g or 8 weeks or >12 months ^1^ mice <18 g or 8 weeks or >12 months ^1^ if age/weight not reported: Animals described as pups, neonatal, juvenile, adolescent, geriatric, aged, old…
Type of interventions	BOLD fMRI of the brain with comparison of different drugs, doses or time points of imaging relative to induction, or anesthetized versus awake for same imaging protocol alteration of physiological parameters: Either deliberately caused by an intervention or closely monitored over time with the explicit intention (mention in abstract) of analyzing the correlation with fMRI signals. Accepted experimental paradigms resting state (rs) central stimulation: Pharmacological, electrical, optogenetic peripheral stimulation: Electrical, mechanical, chemical, thermic, visual, auditory, olfactory, gustatory, visceral	no MRI other (f)MRI modalities other body regions BOLD fMRI of brain tumors BOLD fMRI studies of the brain, but neither comparing anesthetic protocols nor investigating alterations of physiological parameters BOLD fMRI of the brain acquired with animal in vertical position Use of known effects of hypercapnia or hyperoxia to study differences in response to hyperoxic or hypercapnic challenges between experimental groups, but without further characterization of those responses
Definition of anesthetics	all inhalant anesthetics (e.g., isoflurane, sevoflurane, halothane) barbiturates (e.g., thiopental) propofol, alfaxalone ketamine, S-ketamine α-chloralose urethane xylazine, medetomidine, dexmedetomidine acepromazine benzodiazepines opioids if part of a balanced anesthesia protocol	opioids as sole sedative or as intervention in pain studies
Physiological parameters under investigation	arterial blood pressure heart rate respiratory rate partial pressure of CO_2_ partial pressure of O_2_ arterial O_2_ saturation (SpO_2_) pulse distension body temperature hematocrit	all other parameters, e.g., blood glucose levels local temperature of the brain
Interventions to alter physiological parameters	blood withdrawal fluid supplementation pharmacologic manipulation of cardiovascular parameters normobaric changes of inspiratory gas composition apnea changes in body temperature	hyperbaric inspiratory gas
Outcome measures	qualitative measures derived from BOLD signal alone (e.g., list of regions activated by stimulation or belonging to one component in independent component analysis (ICA)) quantitative measures derived from BOLD signal alone (e.g., peak signal change in region of interest (ROI))	correlations of BOLD signal with other modalities (e.g., EEG (electroencephalogram) signal)
Additional criteria		interventions tested on n=1 animal per condition results for comparison of interest not reported in text
Language restrictions	English German French	all other languages

^1^ at the timepoint of imaging.

**Table 2:** Protocols for awake imaging in studies comparing awake and anesthetized imaging.

Dataset	Species	Anesthesia for Fixation	Acclimation Protocol	Comments
Brevard 2003 [77]	Rats	Yes	No	
Duong 2007 [78]	Rats	Yes	No	
Tenney 2003 [79]	Rats	Yes	No	
Airaksinen 2012 [76]	Rats	Yes	No	Paralyzed
Peeters 2001 [80]	Rats	Yes	No	Paralyzed
Lahti 1999 [81]	Rats	Yes	No	
Tsurugizawa 2010 [64]	Rats	Yes	Yes	
Liang 2015b [82]	Rats	Yes	Yes	
Liang 2012a,b, 2013, 2015a, Smith 2017 [83,84,85,86,87]	Rats	Yes	Yes	
Hamilton 2017 [26], Ma 2017 [28]	Rats	Yes	Yes	
Jonckers 2014 [66]	Mice	Yes	Yes	
Chang 2016 [88]	Rats	No	Yes	Trained to voluntarily enter set-up
Desai 2011 [89]	Mice	No	Yes	Chocolate sprinkles for positive reinforcement
Yoshida 2016 [90]	Mice	No	Yes	
Sicard 2003 [91]	Rats	No	No	

## Supplementary Materials

The following are available online at https://www.mdpi.com/2076-2615/11/1/199/s1: Table S1: Overview of included studies covered by this article; Table S2: baseline BOLD signal studies in rats; Table S3: responses to stimulation in rats; Table S4: rsfMRI and functional connectivity studies in rats; Table S5: baseline BOLD signal studies and responses to stimulation in mice; Table S6: rsfMRI and functional connectivity studies in mice; Document S7: Animals Characteristics.
