# Supplementary material for: Systematic Review: Anesthetic Protocols and Management as Confounders in Rodent Blood Oxygen Level Dependent Functional Magnetic Resonance Imaging (BOLD fMRI)—Part B: Effects of Anesthetic Agents, Doses and Timing
Source: Animals (Basel). 2021 Jan 15;11(1):199. doi: 10.3390/ani11010199 (PMC7830239; doi:10.3390/ani11010199)
Supplement: Supplementary file 1 [file animals-11-00199-s001.zip › new_Supplementary Material S7 animal characteristics.pdf]

Supplementary Material S3

Animal characteristics

Data presented here refers to the 83 references which investigated effects of different states of anaesthesia on BOLD fMRI readouts.

Sex

Studies typically investigated male rats or female mice, but the tendency was less pronounced in mice where some studies also explicitly imaged male animals.

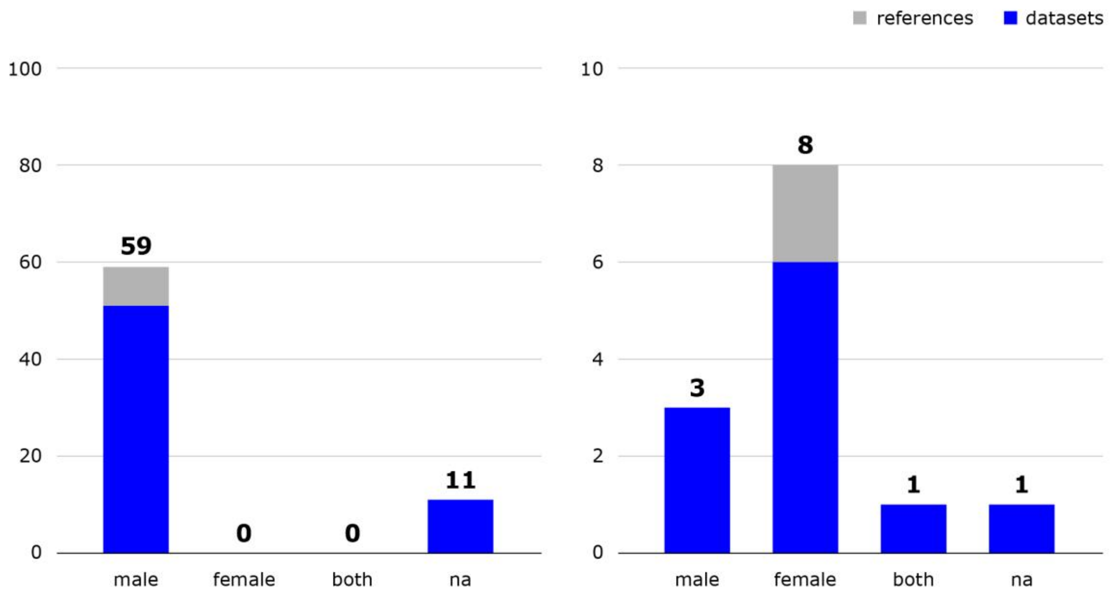

Figure S1. Sex distribution in studies investigating rats (left diagram) and mice (right diagram). Numbers of references and underlying datasets are shown. Male = study used only male animals, female = study used only female animals, both = study used animals of both sexes, na = sex of animals not reported

Strain

An overview about the rat strains used is given in Figure S2. All mouse studies used C57BL/6 mice, and one study additionally investigated BALB/c and I/LnJ [1].

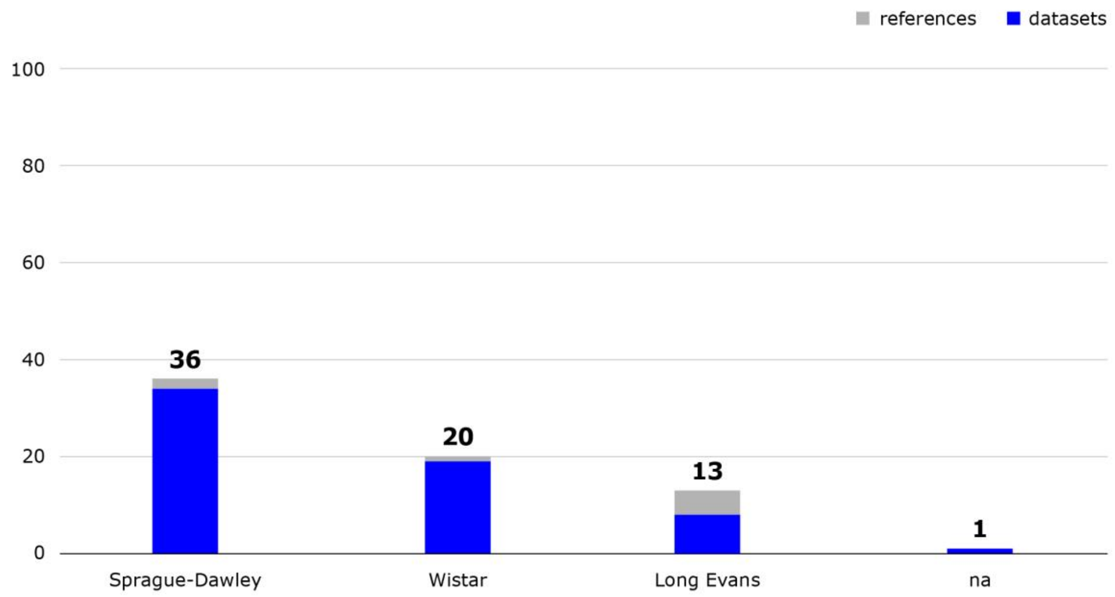

Figure S2. Rat strains used in included studies. Numbers of references and underlying datasets are shown. na = strain not reported

Weight

Weight of the animals was reported in the majority of included studies, only 6 rat datasets and 4 mouse datasets did not report the weight, of which 1 and 3, respectively, reported the age, so that in total 6 references did not report any indicator of animal size.

References

1. Schroeter, A.; Grandjean, J.; Schlegel, F.; Saab, B.J.; Rudin, M. Contributions of structural connectivity and cerebrovascular parameters to functional magnetic resonance imaging signals in mice at rest and during sensory paw stimulation. *J Cerebr Blood F Met* **2017**, *37*, 2368-2382.
