# Supplementary material for: Systematic Review: Anesthetic Protocols and Management as Confounders in Rodent Blood Oxygen Level Dependent Functional Magnetic Resonance Imaging (BOLD fMRI)—Part B: Effects of Anesthetic Agents, Doses and Timing
Source: Animals (Basel). 2021 Jan 15;11(1):199. doi: 10.3390/ani11010199 (PMC7830239; doi:10.3390/ani11010199)
Supplement: Supplementary file 1 [file animals-11-00199-s001.zip › Table S1 overview_abgeglichen mit anderen Tabellen.pdf]

Table S1. Overview of included studies comparing anaesthetic agents with each other or awake imaging, anaesthetic doses and timepoints of imaging. References are grouped by type of fMRI; studies which have examined effects on several types of fMRI are highlighted in dark gray on their second appearance. The anaesthetics under investigation and the type of comparison that was studied for the respective anaesthetic(s) are indicated with an "x". (d) = dexmedetomidine; (S) = S-ketamine; (both) = S-ketamine as well as ketamine; (k/m) = ketamine/medetomidine; (k/x) = ketamine/xylazine; (p) = pentobarbital; (th) = thiobutabarbital.

					Anaesthetics											Comparison			
**Type of experiment**	**Sub-type of experiment**	**Species**	**Publication**	**Re-analysed data of**	Halothane	Isoflurane	(Dex)medetomidine	Isoflurane/(Dex)medetomidine	α-chloralose	Urethane	Propofol	(S-)Ketamine on top	Ketamine/α_2-agonist	Barbiturates	Equithesine	Doses	Time points	Anaesthetic agents	Anaesthetised versus awake
baseline BOLD signal		rats	Abe 2017			x	x									x			
baseline BOLD signal		rats	Gong 2014			x										x			
baseline BOLD signal		rats	Gsell n.d.			x			x									x	
baseline BOLD signal		rats	Liu 2011			x										x			
baseline BOLD signal		rats	Tsurugizawa 2016			x										x			
baseline BOLD signal		rats	Zhurakovskaya 2016							x							x		
baseline BOLD signal		mice	Shah 2016					x									x	x	
baseline BOLD signal		mice	Schroeter 2014			x	x			x	x					x			
phys. parameter change	CO_2 admixture	rats	Brevard 2003			x													x

phys. parameter change	reduced inspiratory O_2	rats	Duong 2007		x													x
phys. parameter change	blood withdrawal	rats	Kalisch 2001	x	x					x							x	
phys. parameter change	apnoea	rats	Kannurpatti 2004						x				x (p)				x	
phys. parameter change	CO_2 admixture	rats	Sicard 2003		x													x
central stim	electrical	rats	Austin 2005	x				x							x	x	x	
central stim	electrical	rats	Chao 2014		x	x (d)											x	
central stim	electrical	rats	Lai 2015		x			x									x	
central stim	optogenetic	rats	Liang 2015b		x													x
central stim	epilepsy model (kainate)		Airaksinen 2012			x												x
central stim	epilepsy model (γ-butyrolactone)	rats	Tenney 2003		x													x
central stim	ketamine	rats	Littlewood 2006a		x						x (both)						x	
central stim	ketamine	rats	Littlewood 2006b		x						x				x		x	
central stim	ketamine	rats	Tomimatsu 2016		x						x						x	
central stim	pharmacological	rats	Liu 2012		x	x			x								x	
central stim	pharmacological	rats	Paasonen 2016b		x	x		x	x				x (th)				x	

central stim	optogenetic	mice	Desai 2011		x													x
periph. stim.	electrical	rats	Brynildsen 2017				x									x		
periph. stim.	electrical	rats	Gsell n.d.		x			x								x	x	
periph. stim.	electrical	rats	Huttunen 2008					x	x								x	
periph. stim.	electrical	rats	Kuo 2005					x					x (p)				x	
periph. stim.	electrical	rats	Lahti 1999							x								x
periph. stim.	electrical	rats	Maandag 2007	x				x									x	
periph. stim.	electrical	rats	Nasrallah 2012			x									x			
periph. stim.	electrical	rats	Nasrallah 2014a		x	x									x			
periph. stim.	electrical	rats	Nasrallah 2014b		x		x									x	x	
periph. stim.	electrical	rats	Pawela 2009			x									x	x		
periph. stim.	electrical	rats	Peeters 2001					x										x
periph. stim.	electrical	rats	Sanganahalli 2009			x		x									x	
periph. stim.	electrical	rats	Sommers 2009		x			x									x	
periph. stim.	electrical	rats	Weber 2006			x		x								x	x	
periph. stim.	mechanical (air puffs)	rats	Chang 2016		x													x
periph. stim.	mechanical (air puffs)	rats	Dashti 2005		x									x			x	
periph. stim.	mechanical (whisker deflection)	rats	de Celis Alonso 2011		x			x									x	
periph. stim.	chemical (formalin sc)	rats	Asanuma 2008		x										x			
periph. stim.	chemical (formalin sc)	rats	Chen 2008		x			x									x	
periph. stim.	visceral - chemical	rats	Tsurugizawa 2010		x			x									x	x
periph. stim.	electrical	mice	Nasrallah 2014c			x									x	x		
periph. stim.	electrical	mice	Schlegel 2015		x	x			x	x							x	

periph. stim.	electrical	mice	Schroeter 2014			x	x			x	x					x		x	
periph. stim.	electrical	mice	Schroeter 2017			x										x			
resting state / fc analysis		rats	Bettinardi 2015										x (k/m)				x		
resting state / fc analysis		rats	Boonzaier 2017			x	x (d)				x							x	
resting state / fc analysis		rats	Brynildsen 2017					x (d)									x		
resting state / fc analysis		rats	Chang 2016			x													x
resting state / fc analysis		rats	Gass 2014				x					x				x	x	x	
resting state / fc analysis		rats	Gill 2017			x										x			
resting state / fc analysis		rats	Grimm 2015	Gass 2014			x					x						x	
resting state / fc analysis		rats	Hamilton 2017			x										x			x
resting state / fc analysis		rats	Herman 2011				x		x									x	
resting state / fc analysis		rats	Hudetz 2015								x					x			
resting state / fc analysis		rats	Hudetz 2016	Hudetz 2015							x					x			
resting state / fc analysis		rats	Hutchison 2010			x							x (k/x)					x	
resting state / fc analysis		rats	Kalthoff 2013			x	x											x	
resting state / fc analysis		rats	Kundu 2014			x		x								x		x	
resting state / fc analysis		rats	Liang 2012a			x													x
resting state / fc analysis		rats	Liang 2012b	Liang 2012a, Zhang 2010 and Liang 2011 (not included)		x													x
resting state / fc analysis		rats	Liang 2013			x													x
resting state / fc analysis		rats	Liang 2015a			x													x
resting state / fc analysis		rats	Liu 2011			x										x			
resting state / fc analysis		rats	Liu 2013a								x					x			

resting state / fc analysis	rats	Liu 2013b			x										x			
resting state / fc analysis	rats	Ma 2017	Hamilton 2017		x										x			x
resting state / fc analysis	rats	Magnuson 2014			x	x (d)										x		
resting state / fc analysis	rats	Nasrallah 2012				x									x			
resting state / fc analysis	rats	Nasrallah 2014a			x	x									x			
resting state / fc analysis	rats	Nasrallah 2014b			x		x									x	x	
resting state / fc analysis	rats	Paasonen 2016a	Paasonen 2016b		x	x		x	x				x (th)			x	x	
resting state / fc analysis	rats	Pan 2011			x										x			
resting state / fc analysis	rats	Pawela 2009				x									x	x		
resting state / fc analysis	rats	Smith 2017	Liang 2012a, Zhang 2010 and Liang 2011 (not included)		x													x
resting state / fc analysis	rats	Tu 2011								x					x			
resting state / fc analysis	rats	Wang 2011			x										x			
resting state / fc analysis	rats	Williams 2010			x	x											x	
resting state / fc analysis	rats	Wilson 2011							x							x		
resting state / fc analysis	rats	Zhurakovskaya 2016							x							x		
resting state / fc analysis	mice	Bukhari 2017	Grandjean 2014		x	x	x										x	
resting state / fc analysis	mice	Grandjean 2014			x	x	x		x	x					x		x	
resting state / fc analysis	mice	Grandjean 2017					x									x		
resting state / fc analysis	mice	Jonckers 2014			x			x	x								x	x
resting state / fc analysis	mice	Mechling 2014				x										x		
resting state / fc analysis	mice	Nasrallah 2014c				x									x	x		
resting state / fc analysis	mice	Schroeter 2017			x		x								x		x	
resting state / fc analysis	mice	Shah 2016			x	x										x	x	
resting state / fc analysis	mice	Wu 2017	Grandjean 2014		x	x	x		x	x					x		x	
resting state / fc analysis	mice	Yoshida 2016				x												x
