# Supplementary material for: Systematic Review: Anesthetic Protocols and Management as Confounders in Rodent Blood Oxygen Level Dependent Functional Magnetic Resonance Imaging (BOLD fMRI)—Part B: Effects of Anesthetic Agents, Doses and Timing
Source: Animals (Basel). 2021 Jan 15;11(1):199. doi: 10.3390/ani11010199 (PMC7830239; doi:10.3390/ani11010199)
Supplement: Supplementary file 1 [file animals-11-00199-s001.zip › Table S2 baseline BOLD signal rats.pdf]

Table S2. Baseline BOLD signal and changes induced by changes in physiological parameters in rats. Summary of main results and classification for figures of all studies addressing effects of anaesthetic protocols on baseline BOLD signal and changes induced by changes in physiological parameters in rats.

Anaesthetics are abbreviated with their first letter(s), "low" and "high" refer to the lower and higher of reported doses, respectively. A vs a = anaesthetised versus awake imaging; sign. = significant; ROI = region of interest; vs = versus; ICA = independent component analysis; ReHo = regional homogeneity; S1 = primary somatosensory cortex; S1FL/HL/BF = forelimb/hindlimb/barrel field area of S1; M(1) = (primary) motor cortex; CPu = caudate putamen; < = smaller/lower; > = larger/higher; ≈ = approximately the same; "…" = cited from the original publication.

**Publication**	**Anaesthetic 1**	**Anaesthetic 2**	**Results**	
**Baseline BOLD signal**				
Abe 2017	Isoflurane 1.5, 2.5%	Medetomidine 0.05 mg/kg sc bolus, CRI 0.1 mg/kg/h for 40 min, then 0.3 mg/kg/h iv	Baseline BOLD signal: increase with higher % I, decrease with higher rate of medetomidine	Dose: yes (I and M)
Gong 2014	Isoflurane 1.8, 3.5%		Baseline BOLD signal: increase with higher % isoflurane	Dose: yes
Gsell n.d.	Isoflurane 2%	α -chloralose 50 mg/kg iv bolus, 40 mg/kg/h iv CRI	90 min after switch to AC baseline BOLD signal 10% lower (significance not reported)	Drugs: partial
Tsurugizawa 2016	Isoflurane 1.5, 2.0, 2.5, 3.0%		BOLD signal intensity in somatosensory cortex: inverse U-shape, i.e. at 1.5% < 2.0 ≈ 2.5% > 3.0% (differences sign.); same pattern when BOLD signal split in "tissue" and "vessel" signal	Dose: yes
Liu 2011	Isoflurane 1.8, 2.0, 2.2%		Baseline BOLD signal: no sign. difference	Dose: no
Zhurakovskaya 2016	Urethane 1.0 g/kg iv; top-up "if found necessary based on (…) reflex(es)"		Baseline BOLD signal: alternating phases of higher and lower baseline signal (in whole brain), supposedly corresponding to fast and slow wave states in EEG.	Time: yes
**Physiological parameter change (intervention) as stimulus**				
Duong 2007 (9, 12 and 17% O_2)	Isoflurane 2%	awake	sign. difference only if paO_2 < 50 mmHg: BOLD signal **decrease** I > awake	A vs a: partial
Brevard 2003 (5 and 10% CO_2)	Isoflurane 2%	awake	BOLD signal **increase** awake > I in cortical as well as in subcortical ROI; in both ROI	A vs a: yes

			signal increase dependent on CO_2 % in awake animals, but under I only in cortical ROI.<br>Rise time of the BOLD signal: awake > I	
Sicard 2003 (5 and 10% CO_2)	Isoflurane 2%	awake	BOLD signal **increase** awake > I<br>(whole brain ROI).<br>Baseline BOLD signal fluctuation: awake > I	A vs a: yes
Kannurpatti 2004 (apnoea when ventilated with room air, 100% O_2 or 2 or 5 % CO_2)	Urethane<br>1.2 g/kg ip	Pentobarbital 60 mg/kg ip	100% O_2, 2 or 5 % CO_2: no difference in amplitude or latency (time to onset and time to peak) of BOLD signal change;<br>Room air: time to maximal signal decrease U > P	Drugs: partial
Kalisch 2001 (repeated blood withdrawal and re-infusion)	Isoflurane 1.1%	Halothane 0.9%<br>Propofol 28.5 mg/kg/h iv	Sign. correlations between blood pressure time course and BOLD signal time course under all three anaesthetics. Authors explicitly **refrain from characterising anaesthetic-specific profiles** due to low animal numbers.	Drugs: no
