# Supplementary material for: Systematic Review: Anesthetic Protocols and Management as Confounders in Rodent Blood Oxygen Level Dependent Functional Magnetic Resonance Imaging (BOLD fMRI)—Part B: Effects of Anesthetic Agents, Doses and Timing
Source: Animals (Basel). 2021 Jan 15;11(1):199. doi: 10.3390/ani11010199 (PMC7830239; doi:10.3390/ani11010199)
Supplement: Supplementary file 1 [file animals-11-00199-s001.zip › Table S3 responses to stimulation rats.pdf]

Table S3. Responses to stimulation in rats. Summary of main results and classification for figures of all studies addressing effects of anaesthetic protocols on responses to stimulation in rats. Publications which re-analysed an existing dataset are highlighted in grey and the publication in which the data set was originally reported indicated in brackets. Note that one datapoint in figures 2, 3 and 5 represents the pooled results of all publications based on one dataset.
Anaesthetics are abbreviated with their first letter(s), “low” and “high” refer to the lower and higher of reported doses, respectively. A vs a = anaesthetised versus awake imaging; sign. = significant; vs = versus; ROI = region of interest; ICA = independent component analysis; ReHo = regional homogeneity; S1 = primary somatosensory cortex; S1FL/HL/BF = forelimb/hindlimb/barrel field area of S1; M(1) = (primary) motor cortex; CPu = caudate putamen; < = smaller/lower; > = larger/higher; ≈ = approximately the same; “…” = cited from the original publication.

**Publication**	**Anaesthetic 1**	**Anaesthetic 2**	**Results**	
**Peripheral electrical stimulation**				
Nasrallah 2012	Medetomidine 0.05 mg/kg ip bolus, 01., 0.2 or 0.3 mg/kg/h ip CRI		no sign. difference in activated areas and % BOLD signal change in S1	Dose no
Nasrallah 2014a	Medetomidine 0.05 mg/kg ip bolus, 01., 0.2 or 0.3 mg/kg/h ip CRI	Isoflurane 1.0, 2.0, 3.0 %	Medetomidine: % BOLD signal change in contralateral S1 not dose-dependent Isoflurane: % BOLD signal change in contralateral S1 lower when I % higher	Dose: M no, I yes
Pawela 2009	Medetomidine no bolus, 0.1 mg/kg/h iv CRI for 120 min, 0.1, 0.15, 0.2 or 0.3 mg/kg/h iv thereafter		Number of activated voxels: slope of stimulation frequency-dependence decreased if secondary rate 0.1 or 0.15, ≈ if secondary rate 0.2 or 0.3. Signal intensity: slope of stimulation frequency-dependence not affected by secondary rate	Dose and time: partial
Brynildsen 2017	isoflurane 0.5-0.75% + dexmedetomidine 0.015 mg/kg ip bolus, 0.015 mg/kg/h sc CRI		Signal intensity of the response in S1: first 30 min < next 2h; 30-90 min ≈ 90-150 min	Time: partial
Nasrallah 2014b	isoflurane 1.3% (+ vehicle for 40 min)	isoflurane 1.3% + medetomidine 0.3 mg/kg/h CRI for 40 min	Isoflurane: activated area unchanged after 40 min; Isoflurane + medetomidine: activated area in S1 larger and %	Drugs: yes, Time: I no

			BOLD signal change higher than at baseline	
Sanganahalli 2009	Medetomidine 0.1 mg/kg/h ip	α-chloralose 46 +/- 4 mg/kg/h ip	Peak responses at different stimulation frequencies (3 Hz with AC, 9 with M), at those frequencies amplitude and shape of BOLD signal response significantly different. Reproducibility of responses (within and between animals) M< AC	Drugs: yes
Weber 2006	Medetomidine 0.05 mg/kg sc bolus, 0.1 mg/kg/h CRI sc	α-chloralose 50 mg/kg iv, 36 mg/kg iv q60 min	Area of activation (mm^2) in S1 M ≈ AC<br>% BOLD signal increase in S1 M ≈ AC<br>Time to first response after bolus M > AC (significance not reported)	Drugs: no; time: M and AC yes
Sommers 2009	Isoflurane 1.2 %	α -chloralose 80 mg/kg iv bolus, 40 mg/kg/h iv CRI	Stereotactic center of activation in S1 I ≈ AC;<br>% BOLD signal change (AUC) I < AC in 1 of 2 stimulation periods	Drugs: partial
Gsell n.d.	Isoflurane 2%	α -chloralose 50 mg/kg iv bolus, 40 mg/kg/h iv CRI	90 min after switch to AC baseline BOLD signal 10% lower (significance not reported); % BOLD signal change “increased (…) and reached a steady state after 80 min” after switch	Drugs partial; time: AC partial
Maandag 2007	Halothane 1%	α -chloralose 45 +/-9 mg/kg/h ip CRI	Activation in S1 detected “more consistently” (significance not reported); number of activated regions other than S1: H > AC; reproducibility of additional activations: H < AC	Drugs: partial
Kuo 2005	α -chloralose 50 mg/kg ip	Pentobarbital 143-173 mg/kg ip	% BOLD signal change: peak at 3 Hz stimulation frequency under both, at 3 Hz (but not other frequencies) AC > H;<br>AC “tighter” cluster of activated voxels in S1 (just images shown, no numbers)	Drugs: partial

Huttunen 2008	α -chloralose<br>60 mg/kg iv, 30 mg/kg iv top-up after 60 min	Urethane 1.25 g/kg ip	Localisation: under both exclusively in S1 and no sign. difference in centre of activation.<br>% BOLD signal increase: sign. different at all stimulation frequencies, responses under AC only with 1-3 Hz, U only 3 Hz and higher.<br>Latency of the response: AC > U (sign.)	Drugs: partial
Peeters 2001	α-chloralose<br>60 mg/kg iv	awake	Size of activated areas AC < awake, in S1FL as well as bilateral somatosensory cortex;<br>Reproducibility of activated areas (volume and localisation) AC > awake;<br>% BOLD signal change AC < awake;<br>significance not reported for any measure	A vs a: partial
Lahti 1999	Propofol<br>48 mg/kg/h iv	awake	% BOLD signal change P < awake;<br>Specificity P > awake (awake “always some evoked activity” in ipsilateral S1, P “little or no evoked signal”)	A vs a: yes
**Peripheral mechanical stimulation**				
Chang 2016	Isoflurane 3%	Awake	Number of activated regions: I < awake<br>Presence of activation in S1: I < awake	A vs a: yes
Dashti 2005	Isoflurane 1.6%	Equithesin (6.5g chloral hydrate, 1.44g pentobarbital, 2.7g magnesium sulphate, 15 ml alcohol, 52.5 ml propylene glycol) 1:1 diluted with saline, 3 ml/kg ip	Presence of response in S1: I > E<br>(all vs. 1/16 scans)	Drugs: yes
de Celis Alonso 2011	Isoflurane 1.0-1.3%	α-chloralose<br>40 mg/kg iv bolus, 10 mg/kg/h iv CRI	Number of activated regions: I < AC;<br>Number of activated voxels (in S1, S2 and ventral posteromedial nucleus nucleus of the	Drugs: yes

			thalamus): I < AC in 2/2 scans;<br>% BOLD signal change in those 3 regions: I < AC in 1/2 scans	
**Peripheral chemical stimulation**				
Asanuma 2008	Isoflurane<br>0.6, 0.8, 1.0, 1.2, 2.0%		Expected response pattern (i.e. early and late response) to formalin injection only at 1.0 and 1.2%	Doses: yes
Chen 2008	Isoflurane, % not reported	α-chloralose<br>70 mg/kg ip	% BOLD signal change: I < AC; no numbers reported	Drugs: partial
**Visceral stimulation**				
Tsurugizawa 2010	Isoflurane 1.5%	α-chloralose<br>50 mg/kg/h iv<br>awake	Activated areas partially overlapping, partially agent-specific (no statistics);<br>occurrence of negative BOLD signal changes: I > AC > awake (none)	Drugs: partial<br>A vs a: I yes, AC partial
**Central electrical stimulation**				
Chao 2014	Isoflurane 1.0-1.3%	Dexmedetomidine<br>0.025 mg/kg sc bolus, 0.05 mg/kg/h sc CRI	Reproducibility of<br>• localisation of activation<br>• activated area<br>• signal amplitude<br>D > I (“extremely unstable” responses under I)	Drugs: yes
Lai 2015	Isoflurane 1.0-1.25%	α-chloralose<br>60 mg/kg iv bolus, 30 mg/kg/h iv CRI	Qulitatively similar frequency-dependence pattern of % BOLD signal change;<br>% BOLD signal change at 3/9 tested stimulation frequencies: I < AC	Drugs: partial
Austin 2005	Halothane<br>0.7, 0.8, 0.9, 1.0, 1.2, 1.5 plus 60% N_2O	α-chloralose<br>80 mg/kg ip, 30 mg/kg ip 30 min after initial bolus and q60 min thereafter	Halothane doses: no sign. difference in activated areas or % BOLD signal change (in stimulated and contralateral motor cortex).<br>α-chloralose: in first 2h same areas activated as under H, afterwards additional areas activated; in motor cortices activated areas AC < H at 1h, from 2-6h post bolus gradual increase, sign. higher	Dose: H no<br>Time: AC yes<br>Drugs: partial

			than at 1h from 3h on and in contralat. Motor cortex sign. higher than under H from 5h on; % BOLD signal change in motor cortices AC = H at 1h, AC > H from 2 and 3h on	
**Optogenetic stimulation**				
Liang 2015b	Isoflurane 1.0-1.15%	Awake	Number of activated areas I < awake<br>% BOLD signal change in activated areas I < awake	A vs a: yes
**Epilepsy models**				
Tenney 2003	Isoflurane 2%	Awake	"BOLD response" I < awake; term not defined, activation maps shown	A vs a: yes
Airaksinen 2012	Medetomidine 0.05 mg/kg sc bolus, 0.1 mg/kg/h sc CRI	Awake	Localisation of activations M ≈ awake; % of seizures causing BOLD response M ≈ awake; awake seizure duration and presence of BOLD response correlated, M not; M shorter interval between seizures associated with higher probability of BOLD response, awake not	A vs a: no
**(S-)Ketamine on top**				
Littlewood 2006a	Isoflurane 1.6%	ketamine 25 mg/kg ip or S-ketamine 25 mg/kg ip	Activated areas: overall similar spatial pattern with activations in multiple regions, but "BOLD contrast" in "hindbrain and frontal regions" K > S-K;<br>signal time courses in selected ROI similar	Drugs: I vs K: yes, K vs S-K: partial
Littlewood 2006b	Isoflurane 1.5%	ketamine 10 or 25 mg/kg sc	Activated areas: multiple areas, spatial pattern differs between doses. Only under higher dose negative correlations of BOLD signal with input function in some regions.	Dose: yes, Drugs: yes
Tomimatsu 2016	Isoflurane 1.5-1.8%	ketamine 10 mg/kg sc	After K positive BOLD signal changes in cortex, hippocampus, CPu and amygdala and negative signal changes in	Drugs: yes

			brainstem, cerebellum and hypothalamus	
**Pharmacological MRI**				
Liu 2012 (Levo-tetrahydro-palmatine)	Isoflurane 1.4-1.6%	Medetomidine 0.1 mg/kg sc bolus, 0.1 mg/kg/h iv CRI	Number of activated regions: I < M < U; Number of regions with negative BOLD signal changes: I < M< U. ANOVA: sign. effect of anaesthetic agent on signal intensity in several regions plus interactions with dose of test-substance	Drugs: yes (between all three)
		Urethane 1.2 g/kg ip		
Paasonen 2016b (nicotine)	Isoflurane 1.3%	Medetomidine 0.01 mg/kg iv bolus, 0.1 mg/kg/h iv CRI	ICA approach: activation detected in similar regions across groups. Anatomically defined ROI: variability in AUC of BOLD signal time courses between regions and anaesthetics some sign. differences	Drugs across all groups: partial
		α-chloralose 60 mg/kg iv bolus; 30 mg/kg iv top-up q60 min		
		Urethane 1.25 g/kg ip (over 15 min)		
		thiobutabarbital 140 mg/kg ip		
