# Supplementary material for: Systematic Review: Anesthetic Protocols and Management as Confounders in Rodent Blood Oxygen Level Dependent Functional Magnetic Resonance Imaging (BOLD fMRI)—Part B: Effects of Anesthetic Agents, Doses and Timing
Source: Animals (Basel). 2021 Jan 15;11(1):199. doi: 10.3390/ani11010199 (PMC7830239; doi:10.3390/ani11010199)
Supplement: Supplementary file 1 [file animals-11-00199-s001.zip › Table S4 functional connectivity rats.pdf]

Table S4. rsfMRI and functional connectivity studies in rats. Summary of main results and classification for figures of all studies addressing effects of anaesthetic protocols on rsfMRI and/or fc in rats. Publications which re-analysed an existing dataset are highlighted in grey and the publication in which the data set was originally reported indicated in brackets. Note that one datapoint in figures 2, 3 and 5 represents the pooled results of all publications based on one dataset. Anaesthetics are abbreviated with their first letter(s), "low" and "high" refer to the lower and higher of reported doses, respectively. A vs a = anaesthetised versus awake imaging; sign. = significant; vs = versus; ROI = region of interest; ICA = independent component analysis; ReHo = regional homogeneity; S1 = primary somatosensory cortex; S1FL/HL/BF = forelimb/hindlimb/barrel field area of S1; M(1) = (primary) motor cortex; CPu = caudate putamen; < = smaller/lower; > = larger/higher; ≈ = approximately the same; "…" = cited from the original publication.

Publi-cation	Anaes-thetic 1	Anaes-thetic 2	Results	Figure
Chang 2016	Isoflurane 3%	Awake	Spatial pattern of temporal correlation between a **seed in S1HL** and all other voxels in the brain: fewer connections under I than awake, under I "mainly localised"; both in resting state and when stimulated with air puffs to mimic chronic pain.<br>**Pairwise correlation coefficients** of 264 anatomically defined regions ("whole brain fc"): qualitatively wider distribution of values in awake animals, FWHM (full width at half maximum) of those correlation coefficients I < awake during rs scan (p= 0.05) and stimulation scan (p < 0.05)	A vs a: yes
Hamilton 2017	Isoflurane 0.5, 1.0, 1.5, 2.0, 2.5, 3.0 %	Awake	**Within thalamocortical and frontoparietal networks** more often reduced than enhanced connectivity under I.<br>**Averaged normalised fc of each voxel:** spatially homogeneous reduction, negative (compared to awake) in all voxels at all doses.<br>**Absolute fc changes** (ROI-ROI as well as between each pair of voxels) negatively correlated with the fc strength in the awake state, and correlation coefficient sign. more negative with higher % isoflurane.<br>**Entropy** of the BOLD signal time course I > awake, sign. increase until 1.5%, then plateau; **mutual information** I < awake, and sign. lower with higher % isoflurane.	A vs a: yes Doses: yes
Ma 2017 (Hamilton 2017)	Isoflurane 0.5, 1.0, 1.5, 2.0, 2.5, 3.0 %	Awake	**Static fc: fc strength between ROI pairs** I < awake and monotonic decrease of static fc with higher % isoflurane, but similar **spatial patterns** across conditions (high spatial correlation coefficients between fc matrices).	A vs a: partial Doses: yes

			**Sliding window approach**: clustering of all matrices into 5 patterns. All occur in all conditions, but one predominantly in awake, one predominantly at 1.5% and one predominantly at 3% isoflurane.	
Liang 2012a	Isoflurane 2%	Awake	**anti-correlation** between the infralimbic cortex and the amygdala: absent under I, present in awake.<br>**Seed in motor cortex**: fc map I < awake, connectivity between bilateral motor cortex I < awake	A vs a: yes
Liang 2012b (Liang 2012a)	Isoflurane 2%	Awake	**Global functional network parameters:**<br>Global clustering coefficient: no sign. diff.<br>Mean shortest path length: no sign. diff.<br>Small worldness: no sign. diff.<br>Modularity: no sign. diff.<br>**Local functional network parameters:**<br>Local clustering coefficient: I < awake in 5/54 ROI<br>Betweenness centrality: I < awake in 3/54 ROI<br>**Community structure:** regions differently organised into modules under I than awake<br>**Fc strength ROI-ROI pairs:** on average I < awake (e.g. in striatum, pallidum, thalamus, cortices), but specific connections also I > awake (e.g. hippocampus, amygdala, hypothalamus).<br>Thalamocortical connectivity in associative vs sensory-motor networks: I < awake in both, but sign. more reduced in associative networks.<br>**Relation physical distance and fc strength:** data not indicative of reduction of “long distance fc” under I	A vs a: partial
Liang 2013 (Liang 2012a)	Isoflurane 2%	Awake	Thalamocortical connectivity (**seed-based** analysis for 8 bilateral **thalamic seeds**): **fc maps** I < awake; averaged **correlation coefficients** “tended” to be lower under I	A vs a: partial
Smith 2017 (Liang 2012a)	Isoflurane 1.5-2.0%	Awake	**Whole brain connectivity of a seed in claustrum**: awake “strong” connections with bilateral cortex and moderate connections with mediodorsal thalamus; under isoflurane significantly decreased connectivity with medial prefrontal cortex and mediodorsal thalamus, but not with other regions.<br>**Seed in insular cortex**: awake strong connectivity to contralateral insular cortex vs under isoflurane strong connectivity with cingulate cortex.	A vs a: yes

			**Seed in prelimbic cortex**: connectivity with claustrum and mediodorsal thalamus I < awake (sign.).	
Liang 2015a (Liang 2012a)	Isoflurane 1.5%	Awake	**Static fc:**<br>**seed in infralimbic cortex**: "consistently weaker" and spatially more confined connectivity under isoflurane; **seed in S1BF** (barrel field) no difference.<br>**Dynamic fc: co-activation patterns (CAP)** for **infralimbic seed:** in first CAP connectivity with limbic regions reduced, second CAP "generally reduced co-activations, third CAP "virtually disappeared" under isoflurane.<br>for **S1BF seed:** all 3 observed also under isoflurane, emergence rates not sign. different	A vs a: partial
Liu 2011	Isoflurane 1.8, 2.0, 2.2%		**Seed in S1FL**: spatial pattern of correlated voxels (**fc map**): "strong correlation over the majority of cortical regions" at 1.8 and 2.0%, mainly at 1.8% additional correlations with CPU and thalamic nuclei.<br>**BOLD signal time course** in S1FL bilaterally synchronous "bumps" at 1.8 and 2.0, but not 2.2%. **Correlation coefficients** of voxels correlated with seed generally lower at 2.2% than at 1.8 and 2.0%;<br>**Magnitude of BOLD signal fluctuation** sign. higher at 1.8 than at 2.0 and 2.2%; interhemispheric correlation sign. higher at 1.8 than at 2.2%.	Doses: yes
Liu 2013b	Isoflurane 1.0, 1.5, 1.8%		**Seeds in S1FL** (left and right) and S1BF (right): spatially less specific **fc maps** at 1.8 than 1.0%. At 1.8% strong correlations in "most cortical and some subcortical regions" vs mainly connections between bilateral S1 at 1.0.<br>**BOLD signal time course** in seed: "bumps" only at 1.8%.<br>**Magnitude of BOLD signal fluctuation** lowest at 1.5%.<br>**Power spectra analysis:** higher power in all frequencies at 1.0 than at 1.5 and 1.8%.<br>**But**: global signal regression made maps at 1.8% specific, while not changing much at 1.0%, and for 1.8% similar results if CC threshold higher and remaining values "expanded".	Doses: yes
Pan 2011	Isoflurane 1.0 – 1.8%		**Interhemispheric connectivity S1FL**: at 1.0 < 1.8 % (sign.)	Doses: yes
Wang 2011	Isoflurane 0.5, 1.0, 2.9%		**Hurst exponent (H)*** of signal time course in left and right S1FL: 0.5 > 1.0 > 2.9%	Doses: partial

			(sign.), approaching 0.5, i.e. white noise, at 2.9%. In 4/5 additional ROI, H also sign. reduced at 2.9% (+/- 1.0%) compared to 0.5% isoflurane and approaching 0.5.<br>**Relative amplitude of BOLD signal fluctuation** (standard deviation of thelow-pass-filtered BOLD signal time series): 0.5 > 2.9%.<br>**Within-ROI connectivity:** at 0.5 > 2.9% (sign.) in all cortical ROI and CPu, not in ventral posterolateral thalamic nucleus.<br>**Interhemispheric fc** between bilateral M1, M2 and CPu: at 0.5 > 2.9% (sign.).<br>**Fc maps**: reportedly reduced number of voxels correlated to the respective seeds at higher % isoflurane (in both hemispheres), but just maps of one animal shown<br><br>***** H > 0.5 positive autocorrelation of the signal "over long time lags",<br>H = 0.5 white noise, H < 0.5 anticorrelation	
Gill 2017	Isoflurane 1.5, 2.0%		33 ROI, if connectivity sign. different between kainate treated and control animals, ROI defined as **"node"**; sign. correlation between nodes as **"edge".**<br>At 2% isoflurane 7 nodes and 6 edges vs 23 and 78 at 1.5% isoflurane.<br>Between-group differences in **global and regional network parameters** (characteristic path length, global efficiency, local efficiency, small-world characteristics, betweenness centrality) consistent at both levels of isoflurane, with the exception of 2/3 parameters for small worldness	Doses: partial
Kundu 2014	Isoflurane 1.0, 1.5, 2.0%	isoflurane 1.5% + medetomidine 0.45 mg/kg sc bolus, 0.2 mg/kg/h iv CRI	**Degree of freedom:** at 1.0 > 1.5 > 2.0% isoflurane; isoflurane and medetomidine ≈ 2% isoflurane<br><br>(high degree of freedom interpreted as a higher amount of structured information within the signal, i.e. desirable)	Doses: I yes<br>Drugs: partial
Brynildsen 2017	isoflurane 0.5-0.75% + dexmedetomidine 0.015 mg/kg ip bolus, 0.015 mg/kg/h sc CRI		**Seed in right orbital frontal cortex**: in first phase (0-30 min) connectivity mainly around seed region, more extended in phases 2 (30-90 min) and 3 (90-15 min). Significant differences between phase 1 and 2 (2 higher correlation with voxels in prelimbic and cingulate cortex) and 1 and 3 (3 higher correlation with voxels in multiple regions), but not 2 and 3.	Time: partial

Nasrallah 2014b	Isoflurane 1.3%	**Followed by** isoflurane 1.3% + medetomidine 0.3 mg/kg/h CRI (no bolus; route not reported) **or** just vehicle infusion	**Seeds in S1FL, Thalamus (Thal) and CPu**: **interhemispheric connectivity** before ≈ after vehicle infusion. After start medetomidine CRI interhemispheric connectivity decreased in S1FL (significant from 25 min after start on) and Thal (significant from 15 min after start on), but not CPu.<br>**Spectral analysis**: under M S1FL correlations in 0.01-0.04 Hz range and Thal correlations in 0.01-0.04 and 0.04-0.07 Hz range sign. reduced.	Drugs: yes<br>Time: I no
Nasrallah 2012	Medetomidine 0.05 mg/kg ip bolus, 01., 0.2 or 0.3 mg/kg/h ip CRI		**Fc map for a seed in S1:** "loss of interhemispheric connectivity" with higher rates<br>**Interhemispheric correlation:** between bilateral S1 and S2 sign. reduced at 0.2 and 0.3 mg/kg/h; between bilateral CPu no difference.<br>**Amplitude of BOLD signal fluctuations:** no sign. difference between left and right S1, S2, CPu or across rates.<br>**Frequency analysis**: S1 and S2 correlations in the 0.01-0.04 Hz and 0.04-0.07 Hz range sign. reduced at 0.2 and 0.3 mg/kg/h, S2 also in 0.07-0.1 Hz range. CPu no sign. difference in any frequency range.	Doses: partial
Pawela 2009	Medetomidine 0.1 mg/kg/h iv CRI for 120 min; 0.1 or 0.3 mg/kg/h iv thereafter (no bolus)		**Regional pairwise correlation coefficient (RPCC) matrices** of sensorimotor network regions**:** if secondary rate constant (0.1 mg/kg/h), correlation coefficients typically decreased and decrease sign. in many cases. If secondary rate increased, increase of correlation coefficient between some regions, but overall similar.	Time: yes<br>Dose: yes
Nasrallah 2014a	Isoflurane 1.0, 2.0, 3.0%	Medetomidine 0.05 mg/kg ip bolus, 01., 0.2 or 0.3 mg/kg/h ip CRI	**Isoflurane:**<br>**Signal time course** in left S1FL: amplitude of fluctuation visibly reduced, manifesting as sign. reduced **total power** of BOLD signal fluctuations: 1 >2 > 3%.<br>**Interhemispheric S1FL connectivity** (ROI-ROI): coherence in 2/5 frequency bands sign. reduced from 1 to 2% and in 5/5 frequency band from 2 to 3% (and 5/5 from 1 to 3%).<br>**Medetomidine:**<br>**Signal time course** in left S1FL: no visible difference in amplitude of signal fluctuation and no significant difference in **total power** of BOLD signal fluctuations.<br>**Interhemispheric S1FL connectivity** (ROI-ROI): coherence in all frequency bands sign. 0.1 > 0.2 and 0.1 > 0.3 mg/kg/h; no	Doses: I yes, M partial

			sign. difference between 0.2 and 0.3 in any frequency band.	
Magnuson 2014	Isoflurane 2% for 30 min ("short") **or** 3 h ("long"; reduced to 1.5% for last 30 min)	**Followed by** dexmedetomidine 0.025 mg/kg sc bolus, 0.05 mg/kg/h sc CRI for 80 min, then 0.15 mg/kg/h)	**Spectral characteristics** of signal time course in S1FL (scan q30 min from 0.75 to 5.75 h post bolus): between groups no sign. difference in spectral centre of mass and maximum location at any time point, but sign. higher maximum power, low-, high- and broadband power in short I group at some timepoints (1.25, 1.75, 2.75h plus 2.25h for broadband power). Within groups, all spectral metrics except centre of mass were sign. different between first and second half of study for long I group. In short I group, only maximum power, high- and broadband power sign. different between first and second half.<br>Qualitatively: power spectra from 3.25h on similar between short and long I groups.<br>**Fc**: **S1-S1 connectivity** based on a seed in S1FL: at 0.75h sign. higher in short I group; long I sign. lower in first half than second. Qualitatively at 2.25h "convergence" of mean S1-S1 connectivity values.<br>**Whole brain connectivity** (averaged correlation between each voxels time course and the averaged time course of all other voxels): at 0.75 and 1.75h sign. higher in short I group, but no difference between first and second half. Qualitatively global connectivity lower values at all time points, similar only at 5.75h.<br>**Spatiotemporal dynamics analysis**: coordinated cortical spatiotemporal dynamic identified 50% or more of scans from 1.75h on in short I group and from 3.25h on in long I group.	Time: I and D partial
Williams 2010	Medetomidine 0.05 mg/kg sc bolus, 0.1 mg/kg/h CRI (route not reported)	**Followed by** Isoflurane 2%	**Power spectral analysis** of BOLD signal time course in a seed in S1: no significant difference between M and I.<br>**Seed in S1:** average correlation values in contralateral S1 I > M (sign.), number of correlated voxels I ≈ M, ratio of correlated voxels in S1 to correlated voxels in the rest of the brain I < M (sign.). Distribution of correlation coefficients: under M peak close to 0, under I "shifted to positive values".<br>**Fc maps** for seeds in S1, S2, CPu: under M distinct maps for each seed and high correlation coefficients located in bilaterally symmetrical areas. Under I no	Drugs: yes

			clear difference between maps for seed in S1 and S2, high correlation values “throughout cortex”.<br>**Pairwise ROI analysis** (left and right S1, S2, CPu): correlation between ipsilateral S1-S2 and ipsilateral S2-CPu sign. lower under M than I.	
Kalthoff 2013	Isoflurane 1.5%	**Followed by** medetomidine 0.05 mg/kg sc bolus, 0.1 mg/kg/h sc CRI	**Independent component analysis (ICA)**: incidence of cortical and striatal networks under M > I; under M but not I, networks consistently segregated into medial, lateral and intermediate components, and striatal components observed at a high incidence.<br>**Fc maps**: under I “widespread cortical or subcortical correlations”, regardless of the chosen seed, vs dominance of connectivity between bilateral homotopic regions under M. After global signal regression (GSR) less widespread correlations under I, but interhemispheric under I < M.<br>**Seed-based correlations**: intrahemispheric cortico-cortical correlation I > M. Correlation strength decreases with distance from seed under M, but not I. Interhemispheric cortico-cortical connectivity after GSR I < M. After GSR interhemispheric striato-striatal connectivity I < M. Cortico-striatal anti-correlation I < M.	Drugs: yes
Boonzaier 2017	Isoflurane % not reported	Dexmedeto-midine dose not reported<br>Propofol dose not reported	**Inter- and intrahemispheric connectivity:** in ANOVA significant effect of anaesthesia; “generally higher values in isoflurane-anesthetized animals as compared to dexmedetomidine and propofol anesthesia”	Drugs:<br>I vs D: yes<br>I vs P: yes
Tu 2011	Propofol 80 or 160 mg/kg ip		**Seed in thalamus, fc map**: connectivity with ipsilateral S1 and contralateral S2 at 80 mg/kg, absent at 160 mg/kg	Doses: yes
Hudetz 2015	Propofol 20 or 40 mg/kg/h iv		**Regional homogeneity** (calculated for sliding windows of 200s): temporal variance of ReHo of selected voxels (criterium: ReHo value > 2 standard deviations from mean) significantly reduced at 40 mg/kg/h.<br>**Cross-correlation between significant ReHo areas**: no sign. difference between doses.<br>**Coincident threshold crossing** (all voxels per image selected which cross a threshold defined as a multiple of each voxel’s SD): temporal variance of BOLD signal of	Doses: partial

			selected voxels significantly reduced at 40 mg/kg/h. Number of unique brain states 81 vs 66 at 20 vs 40 mg/kg/h.<br>CTC variance of BOLD values in 12 anatomically defined ROI: "substantially decreased" at higher doses (38-77%), somatosensory, motor and retrosplenial cortices less affected than visual and parietal cortex and CPu.	
Hudetz 2016 (Hudetz 2015)	Propofol20 or 40 mg/kg/h iv		**Average ReHo in ROI** (ROI = common subset of voxels with ReHo values > 2 standard deviations in all sliding windows): no difference.<br>**ReHo binary maps** when selected voxels classified as above/below defined ReHo threshold at different timepoints: "visually evident" differences in "image patterns" in all animals.<br>**Lempel-Ziv-Complexity***: at 20 > 40 mg/kg/h (sign.)<br>* a proxy for "the amount of non-redundant information contained in a string", which estimates "the minimal number of character sequences […] required to describe the string"	Doses: partial
Liu 2013a	Propofol 20, 40, 60, 80, 100 mg/kg/h iv CRI (no bolus)		**Whole brain fc:**<br>**Fc maps**: most extended at 20 mg/kg/h, at 40 < at 60 mg/kg/h, at 80 "partial recovery", and at 100 mg/kg/h again decrease.<br>**Significantly connected voxel count** (SCVC) per ROI: for 6 cortical ROI SCVC typically decreases at 40, recovers at 60 mg/kg/h, and then decreases again at higher rates. 6 subcortical ROI overall "more stable" SCVC.<br>**K-means clustering of SCVC** at 20, 40 and 60 mg/kg/h: 2 cortical and 2 subcortical clusters of distinct responses.<br>**Regional fc:**<br>For each of the 12 seeds SCVC in target ROI (another seed) determined; ANOVA: sign. dose-dependence in 29 connections.	Dose: yes
Hutchison 2010	Isoflurane 1%	Ketamine 80 mg/kg + Xylazine 10 mg/kg ip	**ICA:** no sign. difference in number of animals with bilateral components; no between-group difference in component-specific power spectra.<br>**Seed-based analysis** (seeds in medial frontal cortex, S1, hippocampus, CPu, thalamus and hypothalamus): bilateral connectivity between seeds and contralateral homotopic region in both groups, interindividual variability in fc maps and thresholds at which	Drugs: no

			interhemispheric connectivity detectable, but no between-group differences.	
Gass 2014	Medetomidine 0.07 mg/kg sc bolus, 0.14 mg/kg/h CRI	**On top** S-ketamine 5, 10, 25 mg/kg sc	**ROI-ROI correlations**: Significant changes in 15/45 pairs of anatomically defined ROI; higher dose = higher increase in correlation coefficient in all except one "prefrontal and cingulate cortex pairs" and "between the infralimbic cortex and both the retrosplenial cortex and posterodorsal hippocampus". Timepoint post injection (15 vs 30 min) no significant effect in any pair. **Seed-based whole brain fc** (7 seeds in hippocampus and prefrontal cortex): for most seeds strongest effect on connectivity to prefrontal regions (increased correlations); in some seeds increase in connectivity stronger at 30 than 15 min post injection.	Time: partial Dose: partial Drug: yes
Grimm 2015 (Gass 2014)	Medetomidine 0.07 mg/kg sc bolus, 0.14 mg/kg/h CRI	**On top** S-ketamine 25 mg/kg sc	**Pairwise correlation between seeds** (left and right prelimbic cortex and left and right hippocampus): after S-ketamine administration significant increase of connectivity in all connections, except between right prelimbic cortex and right hippocampus	Drugs: yes
Bettinardi 2015	Ketamin 60 mg/kg + medetomidine 0.5 mg/kg ip		60-85 min post bolus ("deep anaesthesia") vs 160-185 min post bolus ("light anaesthesia"). **14 bilateral ROI:** Variance of mean BOLD signal across all areas between sliding windows in each phase: light > deep; mean correlation over all ROI-ROI pairs: light > deep; standard deviation of the correlation distribution: light > deep. **mean global synchronization** (assessed by Kuramoto order parameter): light > deep; **functional integration**: light > deep; **functional segregation** (modularity): deep > light. All differences sign. **Emergence of functional networks**: in the light, but not deep phase, five groups of robust connected nodes identified (i.e. pairs of areas consistently correlated across subjects). Between those connected areas correlation increased over time, but not between unconnected areas.	Time: yes
Herman 2011	Medetomidine 0.1 mg/kg/h ip	α-chloralose 40 mg/kg/h ip	**Scaling exponent (fractal index) β** of the "scale-free (fractal) distribution of amplitude power across a frequency range" of BOLD signal fluctuations: Cortical ROI M > AC	Drugs: partial

			Subcortical ROI no difference	
Zhurakovskaya 2016	Urethane 1.0 g/kg iv; top-up "if found necessary based on (…) reflex(es)"		**ROI-ROI connectivity** (motor cortex, somatosensory cortex, hippocampus and thalamus): thalamocortical connectivity in phases of higher baseline signal (supposedly corresponding to fast wave state in EEG) > lower baseline signal (supposedly corresponding to slow wave state in EEG); cortico-cortical connectivity in phases of higher baseline signal < lower baseline signal; connectivity between hippocampus and other regions not affected	Time: partial
Wilson 2011	Urethane 1.5 g/kg ip		**Fc maps**: during supposed slow wave state more extended fc maps.<br>**ROI-ROI connectivity**: sign. increases during slow wave state in all but one pairs involving piriform cortex or dorsal hippocampus.	Time: yes
Paasonen 2016a (Paasonen 2016b)	Isoflurane 1.3%	Medetomidine 0.01 mg/kg iv bolus, 0.1 mg/kg/h iv CRI<br>α-chloralose 60 mg/kg iv bolus; 30 mg/kg iv top-up q60 min<br>Urethane 1.25 g/kg ip (over 15 min)<br>Thiobutabarbital 140 mg/kg ip	**Correlation between nicotine-induced activation (AUC) and average coherence of ROI** (coherence across all ROI-ROI pairs; 91 ROI in total): I and T sign. correlation for pooled cortical, subcortical and “all” regions; M sign. correlation for subcortical and all regions; AC and U no sign. correlations.<br>**Spatial characteristics of fc:** shown in figures, not specifically discussed.<br>**Stability of fc over time**: average coherence not significantly different between 2 timepoints 1h apart for I, M, and a subset of T; significant increase with AC, U and a subset of T	Drugs: overall yes<br>Time: I, M no, T partial, U and AC yes
