# Supplementary material for: Systematic Review: Anesthetic Protocols and Management as Confounders in Rodent Blood Oxygen Level Dependent Functional Magnetic Resonance Imaging (BOLD fMRI)—Part B: Effects of Anesthetic Agents, Doses and Timing
Source: Animals (Basel). 2021 Jan 15;11(1):199. doi: 10.3390/ani11010199 (PMC7830239; doi:10.3390/ani11010199)
Supplement: Supplementary file 1 [file animals-11-00199-s001.zip › Table S5 baseline BOLD signal and responses to stimulation mice.pdf]

Table S5. Baseline BOLD signal and responses to stimulation in mice. Summary of main results and classification for figures of all studies addressing effects of anaesthetic protocols on baseline BOLD signal and responses to stimulation in mice. Publications which re-analysed an existing dataset are highlighted in grey and the publication in which the data set was originally reported indicated in brackets. Note that one datapoint in figures 2, 3 and 5 represents the pooled results of all publications based on one dataset.
Anaesthetics are abbreviated with their first letter(s), “low” and “high” refer to the lower and higher of reported doses, respectively. A vs a = anaesthetised versus awake imaging; sign. = significant; vs = versus; ROI = region of interest; ICA = independent component analysis; ReHo = regional homogeneity; S1 = primary somatosensory cortex; S1FL/HL/BF = forelimb/hindlimb/barrel field area of S1; M(1) = (primary) motor cortex; CPu = caudate putamen; < = smaller/lower; > = larger/higher; ≈ = approximately the same; “…” = cited from the original publication.

Publication	Anaesthetic 1	Anaesthetic 2	Results	
**Baseline BOLD signal**				
Shah 2016	Isoflurane 1.5%	Isoflurane 1.5% + *“on top”* medetomidine 0.3 mg/kg sc bolus, no CRI	raw T2* signal weakest 20 min after bolus administration, significant differences to 1.5% isoflurane for 50 min after bolus.	Drugs: yes Time: yes
Schroeter 2014	Isoflurane 1.0, 1.5%	Medetomidine:<br>low: 0.05 mg/kg iv bolus, 0.1 mg/kg/h CRI;<br>high: 0.1 mg/kg iv bolus followed by 0.2 mg/kg/h iv CRI<br><br>Urethane:<br>low: 1.2 g/kg ip;<br>high: 1.5 g/kg ip<br><br>Propofol:<br>low: 30 mg/kg iv bolus, followed by 120 and later 150 mg/kg/h iv;<br>high: 45 mg/kg iv bolus, followed by 187 and later 225 mg/kg/h iv	Baseline BOLD signal stronger fluctuations at lower dose under I, M and U, but not P<br>(not quantitatively analysed)	Dose: I, M, U partial, P no
**Peripheral electrical stimulation**				
Nasrallah 2014c	Medetomidine 0.3 mg/kg ip bolus,<br>01., 0.6 or 1.0 mg/kg/h ip CRI		Number of activated voxels and % BOLD signal change in S1: no difference.<br>Time to first detectable response: 60 min after bolus with all rates	Dose: no Time: yes
Schroeter 2014	Isoflurane 1.0, 1.5%	Medetomidine:<br>low: 0.05 mg/kg iv bolus, 0.1 mg/kg/h CRI;	Activated regions: all anaesthetics widespread and bilateral responses (including S1), but distinct patterns.	Drugs: overall partial

		high: 0.1 mg/kg iv bolus followed by 0.2 mg/kg/h iv CRI	Size of activated clusters in regions "involved in somatosensory processing": I and M > U and P.<br>Quantitative analysis of signal in contra- and ipsilateral S1HL, Thalamus, S2 and control (visual cortex): I, M, U linear relation between % BOLD signal change and stimulus strength. Average % BOLD signal change per stimulation period "almost identical" in ipsi- and contralateral regions.<br>In S1HL latency P < I < M≈U; initial dip only under M and U; post-stimulus-undershoot only under P. % BOLD signal change decreases over 4 subsequent stimulation periods under I, M, U, but not P. In Thalamus no decrease of % BOLD signal change and no initial dips, but under M "double peak" (initial and slower response to stimulation). In S2 "pronounced negative component" under M and U.<br>Reproducibility: under I and P > M and U<br>Doses (only 2 animals per alternative dose, significance not reported): I, M and U, but not P lower dose higher % BOLD signal change upon activation;<br>M and U lower dose also lower latency to BOLD signal change.	Doses: I, M, U Partial, P no
		Urethane:<br>low: 1.2 g/kg ip;<br>high: 1.5 g/kg ip		
		Propofol:<br>low: 30 mg/kg iv bolus, followed by 120 and later 150 mg/kg/h iv;<br>high: 45 mg/kg iv bolus, followed by 187 and later 225 mg/kg/h iv		
Schlegel 2015 (randomly spaced single pulse instead of block stimulation)	Isoflurane 1.0%	Medetomidine:<br>0.1 mg/kg iv bolus followed by 0.2 mg/kg/h iv CRI	Activated regions: spatially specific activation maps for all four anaesthetics, independent from the model used to generate them.<br>In bilateral S1, Thalamus and control: Signal time courses anaesthetic-specific; I and U both initial dip and post-stimulus undershoot; M and P "pronounced negative and positive signal transients".<br>In Thalamus responses reliably detected under I, M, P, but not U.	Drugs: yes
		Urethane:<br>1.5 g/kg ip		
		Propofol:<br>low: 30 mg/kg iv bolus, 120 and later 150 mg/kg/h iv CRI		
Schroeter 2017	Isoflurane 1.2, 1.5%		Signal amplitude in contra- vs ipsilateral (to stimulus) S1: at both concentrations no difference between hemispheres.<br>% BOLD signal change and interindividual variability: differed across isoflurane levels, but direction not reported and no quantitative measures provided	Dose: partial
**Optogenetic stimulation**				
Desai 2011	Isoflurane 0.7%	awake	Number of activated ROI: I < awake;<br>Activation in "key ROI", i.e. ROI activated in all awake animals: under I 4/5, but % BOLD signal change sign. < awake.	A vs a: partial

			Goodness of fit between BOLD signal time course and canonical haemodynamic response function I < awake.	
