# Supplementary material for: Systematic Review: Anesthetic Protocols and Management as Confounders in Rodent Blood Oxygen Level Dependent Functional Magnetic Resonance Imaging (BOLD fMRI)—Part B: Effects of Anesthetic Agents, Doses and Timing
Source: Animals (Basel). 2021 Jan 15;11(1):199. doi: 10.3390/ani11010199 (PMC7830239; doi:10.3390/ani11010199)
Supplement: Supplementary file 1 [file animals-11-00199-s001.zip › Table S6 functional connectivity mice.pdf]

Table S6. rsfMRI and functional connectivity studies in mice. Summary of main results and classification for figures of all studies addressing effects of anaesthetic protocols on rsfMRI and/or fc in mice. Publications which re-analysed an existing dataset are highlighted in grey and the publication in which the data set was originally reported indicated in brackets. Note that one datapoint in figures 2, 3 and 5 represents the pooled results of all publications based on one dataset. Anaesthetics are abbreviated with their first letter(s), "low" and "high" refer to the lower and higher of reported doses, respectively. A vs a = anaesthetised versus awake imaging; sign. = significant; vs = versus; ROI = region of interest; ICA = independent component analysis; ReHo = regional homogeneity; S1 = primary somatosensory cortex; S1FL/HL/BF = forelimb/hindlimb/barrel field area of S1; M(1) = (primary) motor cortex; CPu = caudate putamen; < = smaller/lower; > = larger/higher; ≈ = approximately the same; "…" = cited from the original publication.

Publi-cation	Anaes-thetic 1	Anaes-thetic 2	Results	Figure
Yoshida 2016	Medetomidine 0.3 mg/kg sc bolus, 0.6 mg/kg/h sc CRI	Awake	**ICA**: cortical and limbic networks (uni- and bilateral) present under both conditions. MANCOVA: connectivity in retrosplenial cortex and hippocampus awake > M (sign.) **Seed based**: 242 ROI in cortex, basal ganglia and hippocampus. Trend for number of ROI-ROI correlations awake > M; for seed in somatosensory cortex sign. pairwise connections only in awake	A vs a: partial
Jonckers 2014	Isoflurane 1.0 %	a-chloralose 120 mg/kg ip<br>Urethane 2.5 g/kg ip<br>Awake	**ICA**: components spatially "not completely overlapping", but in 7 anatomical regions similar components in all groups; in 3 anatomical regions similar components in awake, A and U, but not I; in CPu components in I, A, U, but not awake. **Seed based**: seeds in left M1, left S1 barrel field, left CPu. Cluster size of correlated voxels and peak signal intensity in cluster ("tmax") in both hemispheres. M1: cluster size no sign. diff., tmax left awake > A, U, right I > A, U. S1: cluster size and tmax in both hemispheres awake > I, A, U. CPU: left no sign. diff., right generally small clusters, cluster size and tmax A > U, I. **interhemispheric ROI-ROI connectivity**: M1: ANOVA sign. between group difference, but no comparison sign. in post-hoc Bonferroni. S1: ANOVA sign. between group difference, post-hoc sign. awake > I, A, U and I > A. CPu: very low correlation in all groups, no sign. between-group difference in ANOVA.	A vs a: I, A, U partial Drugs: Partial in all comparisons
Grandjean 2014	Isoflurane 1.0, 1.5 %	Medetomidine low: 0.05 mg/kg iv bolus, 0.1 mg/kg/h CRI;	**Seed-based:** seeds in the sensory cortex (one each in anterior (ant), medial (med), and posterior (post) parietal), dorsal and ventral striatum, limbic system, cingulate cortex, thalamus; results refer to with global signal regression.	Drugs: overall yes Doses: I, M,

		high: 0.1 mg/kg iv bolus followed by 0.2 mg/kg/h iv CRI<br>Urethane low: 1.2 g/kg ip; high: 1.5 g/kg ip<br>Propofol low: 30 mg/kg iv bolus, followed by 120 and later 150 mg/kg/h iv; high: 45 mg/kg iv bolus, followed by 187 and later 225 mg/kg/h iv<br>Isoflurane 0.5% + Medetomidine 0.05 mg/kg iv bolus, 0.1 mg/kg/h CRI	*Drug comparison:*<br>Sensory ant: bilateral correlation under all conditions; sensory med: bilateral correlation under I 1% and P low and M/I; sensory post: bilateral correlation under I 1% and M/I. Ventral and dorsal striatum seed: sign. bilateral correlation under M high and M/I. Limbic system: sign. bilateral correlation under M high and U high. Cingulate cortex: in all groups correlation confined to cingulate cortex, plus anti-correlation in the sensory cortex under P low. Thalamus: no correlation with cortex in any group (without GSR: correlations found under I 1%, P low and U high, but neither M dose).<br>**Interhemispheric correlation**: sensory ant.: I 1%, P low and M/I > M high, M/I > M low; dorsal striatum: M/I and M high > I 1%, P low and U high.<br>*Dose comparison*:<br>generally lower doses "higher FC values and/or better confined regions within the individual networks". M: M low bilateral correlation in ant, med, and post sensory seeds, M high only in ant. I and U: high spatially more extended, less specific correlations to sensory ant. than low.<br>**Frequency analysis:** in cortical seeds highest amplitude at 0.01 Hz for I 1%, P low, U high (1/f distribution), at 0.015 Hz for M/I, at 0.02 Hz for M low and high (no 1/f distribution). **Approximate entropy** (measure for stochastic behaviour of signal) in sensory ant. seed: P low and M/I > M high and low	U, P partial
Wu 2017 (Grandjean 2014)	Isoflurane 1.0%	Medetomidine low: 0.05 mg/kg iv bolus, 0.1 mg/kg/h CRI; high: 0.1 mg/kg iv bolus followed by 0.2 mg/kg/h iv CRI<br>Urethane 1.5 g/kg ip<br>Propofol 30 mg/kg iv	**Regional homogeneity**: **spatial distribution** (ReHo maps): all conditions high ReHo in cortical areas (cingulate cortex, S1, insular cortex). M low, M high, U and P additionally "retained" ReHo in CPu. U and P relatively higher ReHo in thalamus and hippocampus than other conditions; I and M/I also some ReHo in hippocampus.<br>**Voxelwise statistical analysis**: f-test sign. difference between all groups in some areas; pairwise comparisons of ReHo: Somatosensory cortex I,M/I, P > M high; I, M/I > U; Striatum I < M low, M high, M/I, U; "portions of the hypothalamus" M/I < U; "portions of the thalamus" M high < P.	M doses: here no, per dataset partial Drugs: overall yes

		bolus, followed by 120 and later 150 mg/kg/h iv	**ROI analysis**: ReHo in anatomically defined ROI. ANOVA sign. between group differences in cingulate cortex, S1BF, CPu and thalamus, but not insular cortex and hippocampus. Sign. differences in post hoc t-test: cingulate cortex: M/I > M high; S1BF: I and M/I > M high; I and M/I > U; CPu: M high and M/I > I; M low and M high and M/I > P; Thalamus: P > I and M high and M/I; U > M high.	
		Isoflurane 0.5% + Medetomidine 0.05 mg/kg iv bolus, 0.1 mg/kg/h CRI		
Bukhari 2017 (Grandjean 2014)	Isoflurane 1.0%	Medetomidine 0.1 mg/kg iv bolus, 0.2 mg/kg/h iv CRI	**ICA and functional networks**: 16-18 components grouped into 5 networks. **Occurrence of components**: associative cortical network: visual cortex only under M; DMN: prefrontal cortex only under M and dorsal hippocampus only under I; subcortical network: dorsal striatum only under I and lateral striatum only under M/I; extended subcortical network: globus pallidus not under M/I and olfactory tubercle not under M. Lateral cortical network and thalamic network: all components identified in all 3 protocols. **Dual regression of components identified under M and I**: sign. differences, in cortical components strength of coactivation with components I > M, in ventral striatum I < M. **Within and between network connectivity analysis:** distinct patterns under I and M. M/I approximately a "superposition" of the two patterns: "All connections between cortical and subcortical structures observed under medetomidine were preserved for the combination regime except some interactions within the thalamic and the subcortical network", but also " displayed the strong intracortical networks observed for isoflurane."	Drugs: yes
		Isoflurane 0.5% + Medetomidine 0.05 mg/kg iv bolus, 0.1 mg/kg/h CRI		
Schroeter 2017	Isoflurane 1.3, 1.5%	Isoflurane 0.5% + medetomidine 0.05 mg/kg iv bolus, 0.1 mg/kg/h iv CRI	**Seed in S1 (anterior parietal cortex): fc map** differences between strains preserved under all 3 conditions. **amplitude of low frequency signal fluctuations**: affected by anaesthetic condition, but direction not specified, no quantitative information.	Doses: I partial Drugs: partial

Shah 2016	Isoflurane 1.5%	Medetomidine 0.3 mg/kg sc bolus, no CRI	**ICA**: number of regions of the component most similar to default mode network: at 20 min < at 50 min after bolus (increase mainly in posterior regions)<br>**Seed-based**, seed in cingulate cortex: connectivity with retrosplenial cortex and hippocampal regions (i.e. the posterior parts of default mode network) at 20 min < at 50 min after bolus (sign.).	Drugs: yes<br>Time: yes
Grandjean 2017	Isoflurane 0.5% + medetomidine 0.05 mg/kg iv bolus, 0.1 mg/kg/h CRI		**Static fc**: was higher 55 min than 20 min post bolus in several components (barrel field 1 and 2, forelimb, visual and cingulate/retrosplenial cortices, ventral hippocampus), but lower in others (dorsal and lateral striatal component). ICA with 17 ROI.<br>**Dynamic fc**: same atoms ("elementary building blocks of whole-brain dynamic connectivity representing specific dynamic functional states") identified at both timepoints, and only in 3 out of 20 atoms significant increases in atom fluctuation at 55 min	Time: partial
Mechling 2014	Medetomidine 0.3 mg/kg sc bolus, 0.6 mg/kg/h sc CRI		**ICA,** connectivity-matrix between 92 components: sign. change between 30 and 45 min post bolus in 1.4% of pairs; no changes in pairs involving thalamic nuclei.<br>**Graph-theory-based analysis** (components as nodes, sign. connections as edges): no sign. difference in number of functional modules and small-worldness between timepoints.	Time: no
Nasrallah 2014c	Medetomidine 0.3 mg/kg ip bolus, 0.1, 0.6 or 1.0 mg/kg/h ip CRI		**Seed-based interhemispheric connectivity** (6 ROI in S1, S2, visual cortex, CPu, thalamus, hippocampus): under 0.1 mg/kg/h > 1.0 mg/kg/h in 5/6 ROI (except CPu) at 30 and 120 min post bolus; under 0.1 ≈ 0.6 mg/kg/h at 30 min, 0.1 > 0.6 in thalamus at 120 min.<br>**Frequency analysis in S1**: mean peak frequency not sign. different between doses.<br>**Note:** the slower and weaker the response to stimulation (between the rs scans), the weaker the bilateral S1 connectivity	Dose: partial<br>Time: partial<br><br>Interaction between dose and time
